# ABCB5^+^ mesenchymal stromal cells therapy protects from hypoxia by restoring Ca^2+^ homeostasis in vitro and in vivo

**DOI:** 10.1186/s13287-022-03228-w

**Published:** 2023-02-09

**Authors:** Kaixuan Yan, Jiaxing Zheng, Mark Andreas Kluth, Lin Li, Christoph Ganss, Benito Yard, Richard Magdeburg, Markus H. Frank, Prama Pallavi, Michael Keese

**Affiliations:** 1 Department of Surgery, Medical Faculty Mannheim, Heidelberg University, Mannheim, Germany; 2 European Center of Angioscience (ECAS), Medical Faculty Mannheim, Heidelberg University, Mannheim, Germany; 3 TICEBA GmbH, Heidelberg, Germany; 4 RHEACELL GmbH & Co. KG, Heidelberg, Germany; 5 V Department of Medicine, Medical Faculty Mannheim, Heidelberg University, Mannheim, Germany; 6 Department for General and Visceral Surgery, Theresienkrankenhaus Mannheim, Mannheim, Germany; 7 Department of Dermatology, Brigham and Women’s Hospital, Harvard Medical School, Boston, MA USA; 8 Transplant Research Program, Boston Children’s Hospital, Harvard Medical School, Boston, MA USA; 9 Harvard Stem Cell Institute, Harvard University, Cambridge, MA USA; 10 School of Medical and Health Sciences, Edith Cowan University, Perth, WA Australia; 11 Department of Surgery, University Hospital Mannheim, Theodor-Kutzer-Ufer 1-3, 68161 Mannheim, Germany

**Keywords:** Peripheral artery disease, Mesenchymal stromal cells, Stem cell therapy, Hypoxia, Calcium ion, Endothelial cells, Angiogenesis

## Abstract

**Background:**

Hypoxia in ischemic disease impairs Ca^2+^ homeostasis and may promote angiogenesis. The therapeutic efficacy of mesenchymal stromal cells (MSCs) in peripheral arterial occlusive disease is well established, yet its influence on cellular Ca^2+^ homeostasis remains to be elucidated. We addressed the influence of ATP-binding cassette subfamily B member 5 positive mesenchymal stromal cells (ABCB5^+^ MSCs) on Ca^2+^ homeostasis in hypoxic human umbilical vein endothelial cells (HUVECs) in vitro and in vivo.

**Methods:**

Hypoxia was induced in HUVECs by Cobalt (II) chloride (CoCl_2_) or Deferoxamine (DFO). Dynamic changes in the cytosolic- and endoplasmic reticulum (ER) Ca^2+^ and changes in reactive oxygen species were assessed by appropriate fluorescence-based sensors. Metabolic activity, cell migration, and tube formation were assessed by standard assays. Acute-on-chronic ischemia in Apolipoprotein E knock-out (ApoE^−/−^) mice was performed by double ligation of the right femoral artery (DFLA). ABCB5^+^ MSC cells were injected into the ischemic limb. Functional recovery after DFLA and histology of gastrocnemius and aorta were assessed.

**Results:**

Hypoxia-induced impairment of cytosolic and ER Ca^2+^ were restored by ABCB5^+^ MSCs or their conditioned medium. Similar was found for changes in intracellular ROS production, metabolic activity, migratory ability and tube formation. The restoration was paralleled by an increased expression of the Ca^2+^ transporter Sarco-/endoplasmic reticulum ATPase 2a (SERCA2a) and the phosphorylation of Phospholamban (PLN). In acute-on-chronic ischemia, ABCB5^+^ MSCs treated mice showed a higher microvascular density, increased SERCA2a expression and PLN phosphorylation relative to untreated controls.

**Conclusions:**

ABCB5^+^ MSCs therapy can restore cellular Ca^2+^ homeostasis, which may beneficially affect the angiogenic function of endothelial cells under hypoxia in vitro and in vivo.

**Supplementary Information:**

The online version contains supplementary material available at 10.1186/s13287-022-03228-w.

## Background

Peripheral artery disease (PAD) is characterized by decreasing in the supplying blood to the upper or lower limbs because of the narrowing or blockage of the arteries [1]. Our research focuses on the PAD that affects the leg(s). Hypoxia in the muscle and soft tissue of the leg(s) due to poor perfusion is one of the main characteristics of the pathogenesis of PAD [2]. Exposure to hypoxia activates the angiogenic function of endothelial cells (ECs) [3–5]. This process plays a pivotal role in the pathophysiology of various diseases such as cerebral ischemia, myocardial infarction, and PAD [6, 7]. However, the natural course and the relatively poor prognosis of PAD indicate the lower capillary density induced by hypoxia [8, 9].

Over the past two decades, stem cells have emerged as an alternative for the treatment of patients with PAD. Reduction in resting pain, pain-free walking time and ulcer healing were reported after autologous stem cells treatment of critical limb ischemia in the patients [10–12]. However, autologous stem cells treatment is still facing some challenges that hamper its implementation as a mainstay of treatment. Because the majority of the PAD patients are elderly and often present other co-morbidities, harvesting autologous stem cells from such patients is tedious as the amount and potency of stem cells is generally reduced in elderly donors. It should also be underscored that autologous stem cells treatment in PAD patients did not reduce rates of limb amputation [13, 14]. These shortcomings of autologous stem cells, therefore, warrant further studies on the efficacy of other types of stem cells.

The MSCs were normally isolated from the bone marrow or adipose tissue of people of different ages and morbidities which may lead to the inhomogeneity of functional properties of MSCs while the ATP-binding cassette subfamily B member 5 positive mesenchymal stromal cells (ABCB5^+^ MSCs) were manufactured by TICEBA GmbH from strict health skin tissue donations of donors whose age ≤ 50 years old, which could guarantees reliable and reproducible cell quality and functionality [15]. ABCB5^+^ MSCs express mesenchymal lineage markers (CD90, CD105 and CD73) [16], lack hematopoietic lineage markers CD34, CD14, CD20 and CD45; and show significantly increased adipogenic, osteogenic and chondrogenic differentiation potential [16]. In addition to this, ABCB5^+^ MSCs exhibit immunomodulatory effects through interaction with macrophages [17] and regulatory T lymphocytes [18]. They exhibit a strong paracrine capacity including interleukin 1 receptor antagonist [16, 17] and under hypoxia ABCB5^+^ MSCs secret vascular endothelial growth factor (VEGF) and can trans-differentiae into CD31^+^ ECs [16]. ABCB5^+^ MSCs suppress reactive oxygen species (ROS) release and extracellular trap formation from activated human peripheral neutrophils [19]. Thus, the immunomodulatory, paracrine as well as trans-differentiation capabilities of ABCB5^+^ MSCs make them an ideal stem cell therapy candidate to restore the tissue integrity after hypoxic injury in PAD.

Under physiological conditions, hypoxia or low oxygen tension favors the stabilization of hypoxia inducing factor (HIF)-1α that together with HIF-1β forms the active HIF-1 complex, required for the expression of a number of pro-angiogenic genes [3–5, 20, 21]. In PAD patients, however, this neovascular response seems to be insufficient for adequately reperfusion the ischemic tissue. Apart from angiogenesis, chronic hypoxia also adversely affects the mitochondrial-endoplasmic reticulum (ER) crosstalk by changing ROS and Ca^2+^ homeostasis [22–28], thereby favoring cell dysfunction, apoptosis and end organ-damage [6, 29].

Ca^2+^ homeostasis is essential to regulate a wide variety of cellular processes including angiogenesis [34]. It involves a tight regulation of uptake and release of Ca^2+^ into and from intracellular organelles such as the ER as well as a controlled influx from the extracellular environment [30, 31]. Arnould et al. and Suresh et al. both found that hypoxia could induce the extracellular Ca^2+^ influx that leads to an increase in cytosolic (Cyto) Ca^2+^ concentration [22, 23]. The hypoxia could liberate Ca^2+^ from ER by the generation of mitochondrial ROS [23, 32]. Paul T and his colleagues found that the ER Ca^2+^ release was needed during the process of angiogenesis [24]. However, the Cyto Ca^2+^ overloading induced by hypoxia could lead to angiogenic dysfunction and apoptosis [26–28]. The damaging effect of hypoxia on ER Ca^2+^ homeostasis in ECs during angiogenesis and the influence of ABCB5^+^ MSCs on Ca^2+^ homeostasis in hypoxic ECs have not been extensively studied. In the present study, we test the hypothesis that ABCB5^+^ MSCs yield a beneficial effect on angiogenesis by restoring Ca^2+^ homeostasis in the ECs. We tested this hypothesis by addressing if ABCB5^+^ MSCs alleviate the overloading of Cyto Ca^2+^ and restore ER Ca^2+^ in hypoxic HUVECs. We further evaluated the effect of ABCB5^+^ MSCs on hypoxia-induced ROS burden in HUVECs. Finally, we tested if ABCB5^+^ MSCs restore ER Ca^2+^ using the Sarco-/endoplasmic reticulum ATPase 2a (SERCA2a)- Phospholamban (PLN) axis in both in-vitro cell models as well as in-vivo hind limb ischemia model in Apolipoprotein E knock-out (ApoE^−/−^) mice, which model chronic on acute hypoxia.

## Materials and methods

### Cell lines and cell culture

Human umbilical cord vein endothelial cells (HUVECs) used in this project were isolated from human umbilical cords which were kindly provided by the Department of Obstetrics and Gynecology, University Medical Center Mannheim, University of Heidelberg, Mannheim, Germany. The isolation procedure was approved by the local ethics committee (Ethikkommission II der Medizinische Fakultät Mannheim AZ 2015-518N-MA). The HUVECs isolation was performed as previously described [33]. The identity of the isolated cells was confirmed by immunofluorescence staining for classical endothelial markers-CD31, von Willebrand factor (vWF) and VE-Cadherin. The HUVECs were cultured on 1% gelatin-coated (Sigma-Aldrich, Germany, 9000-70-8) culture flasks in endothelial cell growth medium (Provitro, Germany, 2011101) supplemented with 5% fetal bovine serum (FBS, Gibco, Brazil, 10270106) and 1% penicillin and streptomycin (P/S, Sigma-Aldrich, Germany, 2011101).

ABCB5^+^ MSCs were manufactured by TICEBA GmbH (Heidelberg, Germany) as described before [15]. ABCB5^+^ MSCs were cultured in Ham’s F-10 Medium (Biochrom GmbH, Germany, F0715) with 10% of FBS, 1% of P/S and 2 mM L-glutamine (Roth, Germany, 56-85-9).

ABCB5^+^ MSCs and HUVECs were all incubated at 37 °C in a humidified incubator with 5% CO_2_. The medium was changed every two days. HUVECs between passages 2–5 and the ABCB5^+^ MSCs between passages 1–3 were used for all experiments [15].

### In-vitro hypoxia and co-culture

In-vitro hypoxia was modeled by treating HUVECs with 80 μM Cobalt (II) chloride (CoCl_2_, Sigma-Aldrich, Germany, C8661, Additional file 1: Fig. S1A–D) or 120 μM Deferoxamine (DFO, Sigma-Aldrich, Germany, D9533, Additional file 2: Fig. S2A–D) for 4 h. In another experimental condition to irreversibly inhibit SERCA2a, HUVECs were treated with 3 μM Thapsigargin (TG, Sigma-Aldrich, Germany, T9033, Additional file 3: Fig. S3A) for 4 h.

In this research, two kinds of therapeutic approaches for the application of ABCB5^+^ MSCs as previously reported [34, 35]. The first approach is to coculture the HUVECs coculture with ABCB5^+^ MSCs in the coculture system (ABCB5^+^ MSC-CO). The second approach is to use the ABCB5^+^ MSCs- conditioned medium to treat the HUVECs (ABCB5^+^ MSC-CM). For the ABCB5^+^ MSC-CO system, 0.5 × 10^4^ ABCB5^+^ MSCs per cm^2^ were seeded in cell culture inserts fit for 6-well (4.5 cm^2^/insert, Greiner Bio-One, Germany, 657640) and 24-well plates (0.3 cm^2^/insert, Greiner Bio-One, Germany, 657640). 2 × 10^4^ HUVECs per cm^2^ were seeded in gelatin-coated 6-well and 24-well plates (9.6 cm^2^/well and 1.9 cm^2^/well) as previously reported [36–38]. The co-culture was performed in a 1:1 mix of HUVECs and ABCB5^+^ MSCs medium. ABCB5^+^ MSC-CM was collected as previously reported [35]. The conditioned medium was aliquoted and stored at − 80 °C for later use. In all the in-vitro cell culture experiments HUVECs were first treated with either CoCl_2_ or DFO or TG for 4 h and afterward co-cultured with ABCB5^+^ MSCs or cultured in ABCB5^+^ MSC conditioned medium with the continued presence of CoCl_2_ or DFO or TG.

### MTT assay

MTT (3-(4, 5-dimethyl thiazolyl-2)-2, 5-diphenyltetrazolium bromide, Sigma, USA, M2128) assays were performed to detect the metabolic activity of HUVECs. Briefly, log-phase HUVECs were seeded into a gelatin-coated 24-well plate (1 × 10^6^ cells/well). 24 h post-seeding cells were first treated with either 80 μM CoCl_2_ or 120 μM DFO or 3 μM TG for 4 h. Thereafter, HUVECs were either cultured in ABCB5^+^ MSC-CM or co-cultured with ABCB5^+^ MSCs in presence of 80 μM CoCl_2_, 120 μM DFO or 3 μM TG for another 24 h. Afterward, 20 μL of the MTT dye (0.5 mg/mL) was added to each well and cells were incubated further for a period of 4 h. The formazan salts were dissolved with 100 μL freshly prepared MTT solvent constituting 40% of Dimethyl sulfoxide (DMSO, Roth, Germany, HN47.1), 40% of 10%-sodium dodecyl sulfate (SDS, Roth, Germany, 0183.3), 20% of DPBS, and 1.2% of Acetic acid (Roth, Germany, 6755.1) and absorbance (wavelength 540 nm, reference length 630 nm) was recorded with a Tecan multimode microplate reader (Spark, Switzerland).

### Scratch assay

Scratch assays were performed to evaluate the cell migration property of HUVECs. To this end, 1 × 10^6^ HUVECs per well were seeded into the 1% Gelatin-coated 24-well plate to establish a single cell monolayer in medium supplemented with 3% FBS. After overnight incubation, the cells were first treated with either 80 μM CoCl_2_ or 120 μM DFO or 3 μM TG for 4 h. For CoCl_2_, DFO and TG treated cells monolayer was scratched along a straight line by using a 200 μL pipette tip. For the ABCB5^+^ MSC groups, HUVECs were either co-cultured with the ABCB5^+^ cells or cultured in ABCB5^+^ CM after the 4 h treatments with CoCl_2_ or DFO or TG. The HUVECs treatment with CoCl_2_ or DFO or TG during ABCB5^+^ MSC therapy was continued. The photos of the monolayer were taken at 0 h, 6 h, 12 h, and 24 h by an inverted microscope (Axiovert 200 M; Zeiss, Jena, Germany). The gap area was quantitatively evaluated using ImageJ software (version 1.52, Bethesda, USA).

### Tube formation assay

The HUVECs tube formation assay was employed as reported previously [39]. Briefly, HUVECs were first treated with 80 μM CoCl_2_, 120 μM DFO or 3 μM TG for 4 h. Subsequently, cells were harvested and diluted to 2.5 × 10^4^ cells/mL in medium supplemented with 1% FBS and seeded into the matrigel basement membrane matrix phenol red-free (Corning, USA, 356,237) pre-coated 24-well plates (1 × 10^5^ cells/well). Afterward, HUVECs were either co-cultured with ABCB5^+^ MSCs or cultured in ABCB5^+^ MSC-CM, and treated with 80 μM CoCl_2_, 120 μM DFO or 3 μM TG was continued for 24 h. Images were taken by an inverted microscope after 6 h incubation. The number of HUVECs meshes and junctions were analyzed by the angiogenesis analyzer plugin for ImageJ.

### Cloning, virus production and transduction

#### D1ER

D1ER, genetically encoded fluorescence resonance energy transfer (FRET) based ER Ca^2+^ biosensor which contains enhanced yellow fluorescent protein (YFP: Ex 514 nm/Em 527 nm) and enhanced cyan fluorescent protein (CFP: Ex 430 nm/Em 474 nm) [40] was used to study changes in ER Ca^2+^ [40]. It was synthesized by Genewiz from Merck Sigma Aldrich in pUC57 with BamHI and XbaI restriction sites and further cloned into pHR'SIN-cPPT-SEW via BamHI and XbaI restriction sites. Lentivirus particles were produced as previously described [41, 42] and HUVECs were transduced. The ratio of FRET was calculated by dividing CFP-intensity by YFP-intensity both detected from D1ER (FRET ratio = YFP/CFP). This ratio represented the dynamic changes in ER Ca^2+^ concentration [43].

#### roGFP3

It was used to monitor the dynamic changes of ROS signaling [44]. Lentivirus encoding roGFP3 were generated and HUVECs were transduced as previously described [41].

The stable expression of D1ER and roGFP3 in HUVECs was verified over passages by qualitative assessment of fluorescence intensity. Up to three passages after transduction, no evident expression changes were observed.

### Measurement of ER Ca^2+^

HUVECs transduced with the D1ER sensor were seeded into the 1% gelatin-coated 24-well-plates (3.8 × 10^4^ cells per well). 24 h post seeding cells were treated with 80 μM CoCl_2_, 120 μM DFO and 3 μM TG for 4 h. Thereafter, cells were either co-cultured with ABCB5^+^ MSCs or cultured in ABCB5^+^ MSC-CM for 24 h. The fluorescence intensity of CFP and YFP were detected by a Tecan multimode microplate reader every 2 h. In addition to live cell measurements, ER Ca^2+^ was also measured by confocal microscopy. To this end HUVECs transduced with D1ER sensor were seeded into the 1% gelatin-coated 15 mm coverslips in 24-well-plates (3.8 × 10^4^ cells per well) and the experiment was set up as described above. After the 24 h treatment, cells were fixed with 4% paraformaldehyde (PFA) and imaged by an SP5 microscope system (Leica, Germany) to detect the fluorescence emitted by YFP and CFP. The images were analyzed by NIH ImageJ version 1.52 software to compare the FRET ratio among the different groups.

### Measurement of Cyto Ca^2+^

Fura-2-acetoxymethyl ester (Fura 2-AM, Biotium, USA, 50,033) was used to detect changes in the Cyto Ca^2+^ as previously described [18]. These experiments were performed in two formats.

In the first experiment, 3.8 × 10^4^ HUVECs seeded per well in 24-well plates were treated with 80 μM CoCl_2_ or 120 μM DFO or 3 μM TG or 100 μM hydrogen peroxide (H_2_O_2_) in HHBSS without Ca^2+^ for first 2 h. Afterward buffer was changed to HHBSS with Ca^2+^ for the following 2 h. 100 μM Ionomycin (Ion, Sigma, Germany, I9657) was also used as the positive control.

In the second experimental setup, cells were treated by 80 μM CoCl_2_, 120 μM DFO, and 3 μM TG for 4 h. After that, they were either co-cultured with ABCB5^+^ MSCs or cultured in ABCB5^+^ MSC-CM for 24 h, under treatment with 80 μM CoCl_2_, 120 μM DFO or 3 μM TG for 24 h, separately. 100 μM Ion and 100 μM H_2_O_2_ served as the positive control. The dynamic changes of Cyto Ca^2+^ were detected by monitoring the fluorescence (Em 535 nm) from Fura 2-AM excited by wavelengths of 340 nm and 380 nm on the multimode microplate reader. The fluorescence intensity of Fura 2-AM was monitored by a Tecan multimode microplate reader every 2 h.

### Measurement of ROS signal

HUVECs expressing roGFP3 were seeded into 24-well-plates (1 × 10^6^ cells/well). One day post seeding cells were first treated with either 80 μM CoCl_2_ or 120 μM DFO for 4 h. Afterward, HUVECs were either co-cultured with ABCB5^+^ MSCs or cultured in ABCB5^+^ MSC-CM for 24 h, and treatment with 80 μM CoCl_2_, 120 μM DFO or 3 μM TG was continued for 24 h. HUVECs treated with 100 μM H_2_O_2_ served as the positive control. The dynamic changes in the ratio of fluorescence intensity emitted by roGFP3were monitored on the Tecan multimode microplate reader as previously described [41].

### PAD ApoE^−/−^ mouse model

All experimental procedures on animals were performed according to the EC guideline EC 2010/63/EU and have been approved by the local German government authority (35-9185.81/G[1]239/18).

8-week-old male ApoE^−/−^ mice with the C57BL/6J background were purchased from Charles River Laboratories. The animals were housed in a 12 h light/dark cycle and fed a Western Diet (5% cholesterol and 21% fat) for 12 weeks with free access to food and water.

Mice were anesthetized with a subcutaneous injection (S.C.) of a mixture of Midazolam (5 mg/kg, Ratiopharm, Germany, 44856.01.00), Medetomidine (0.05 mg/mL/kg, Cayman Chemical, USA, 128366-50-7) and fentanyl (0.5 mg/kg, Cayman Chemical, Germany, 437-38-7) before all surgical procedures. Double ligation of the right femoral artery (DLFA) was performed using Ethicon 7-0 sutures (PROLENE, Germany, 8776H) as described previously to simulate an acute-on-chronic model of PAD [45]. Immediately after DLFA, 100 μL ABCB5^+^ MSCs (1 × 10^8^ cells/mL) suspension was injected into 5 different muscle localizations of the right hind limb (20 μL each localization) in the treatment group (*n* = 12). Animals in the control group (*n* = 12) received 100 μL normal saline in the same way. For the postoperative analgesia, butorphanol (1 mg/kg, S.C., Q8h, Dolorex, Germany, 42408-82-2) was given to the mice 24 h after DLFA. Drinking water was supplemented with metamizole (24 mg/5 mL of water, corresponding to a dose of 200 mg/kg 4 times daily) to maintain the analgesic effect for 2 days following the DLFA procedure. On the 1st day after the operation, magnetic resonance imaging (MRI) scans of the bilateral hind limbs were respectively taken to document the perfusion-situation of the proximal and distal femoral artery (FA). 9.4 T scanner (Bruker BioSpec 94/20 AVIII, Bruker Biospin MRI GmbH, Ettlingen, Germany) as described in our previous research [45].

### Functional evaluation and follow-up

To assess the functional recovery of the hind limb movement, mice were visually examined at the 0th, 1st, 3rd, 5th, and 7th days by functional scoring using the Tarlov scale as described previously [46, 47].

### Plasma and tissue collection

The animals were sacrificed 7 days after the DLFA. Blood was collected from the inferior vena cava. Plasma was prepared by centrifuging the blood at 1000 rpm for 10 min at 4 °C and stored at − 80 °C for biochemical analysis. Both sides of the fresh Vastus Lateralis (VL) and Gastrocnemius (GM) were rapidly removed after perfusion with 4% PFA and kept at − 80 °C for further analysis.

### Histological evaluation

The histological analysis of bilateral GM and aorta with Hematoxylin & Eosin (HE) and Immunohistochemistry (IHC) of CD31 and vWF staining were performed on all mice as described in [45]. ImageJ software (version 1.52, Bethesda, USA) was used to estimate the percentage of the microvascular area (CD31 or vWF positive area) in randomly selected 5 fields of view (× 40).

To estimate the plaque burden in the vessels, the thoracoabdominal aorta was removed, fixed overnight in 4% PFA and paraffin-embedded sections (4 μm) were derived. To determine the atherosclerotic burden (AS) in the artery, sections were stained for HE, and the progression stage of AS lesions were determined as previously described [48]. All the sections were inspected by conventional light microscopy.

### Lactate dehydrogenase (LDH) assay

The relative levels of LDH in the VL tissue were detected using an LDH kit (Abcam, Germany, ab197000) following the manufacturer’s guidelines. Briefly, 40 mg tissue was homogenized using a T18 digital homogenizer (IKA, USA) at the speed of 2.5 × 10^4^ rpm for 30 s in protein isolation buffer containing 20 mM Tris–HCl, 150 mM NaCl, 5 mM EDTA, 1% Triton X-100, 0.5% sodium deoxycholate, 1 μM dithiothreitol (DTT), protease and phosphatase inhibitors and incubated on ice for 10 min. Tissue lysates were subsequently centrifuged for 5 min at 4 °C at 1 × 10^5^ g. Supernatant samples were collected and added to 96-well-plate (50 μL each well). Hereafter, a 50 μL reaction mix supplied by the kit was added to each sample and the fluorescence intensity at 535/587 nm (Ex/Em) was immediately measured using a Tecan multimode microplate reader.

### Myoglobin (Mb) ELISA

Mouse Mb sandwich ELISA Kit (Abcam, Germany, ab210965) was used to determine levels of Mb in the VL tissue lysate according to the manufacturer’s protocol. Briefly, 50 μL of tissue lysate (previously described in the LDH assay) followed by 50 μL of the Antibody Cocktail was added to each well of the 96-well-plate and incubated at room temperature for 1 h on a shaker (400 rpm). Afterward, the plate was washed thrice with 350 μL of wash buffer. 100 μL of 3,3′,5,5′-Tetramethylbenzidine development solution was then added to each well and the plate was incubated for 10 min in dark. Finally, the stop solution was added (50 μL each well) and optical density was measured at 450 nm on a multimode microplate reader.

### Cholesterol, triglycerides and creatine kinase in plasma

Cholesterol, triglycerides and creatine kinase (CK) were determined in the plasma of each mouse using Cobas c 311 analyzers (Roche, Switzerland). The analysis was performed as per the manufacturer’s protocol.

### Western blot (WB)

Protein expression was determined by performing WB. Total protein isolated from the HUVECs and mice hind limb GM were mixed with loading buffer (Bio-Rad, Germany, 161-0767) and distilled water to obtain a concentration of 1 μg/μL and denatured by incubation at 100 °C for 10 min. The denatured protein (25 μg) was loaded on the 10% SDS-PAGE acrylamide gels and then electrophoresed at 230 V for 1 h. Protein samples were transferred onto polyvinylidene fluoride membranes (PVDF, Bio-Rad, Germany, 1620177) by using a Turbo blot (Bio-Rad, Germany). Hereafter, the PVDF membrane was blocked for 1 h in Tris-buffered saline with 0.1% Tween 20 (TBST) containing 5% nonfat milk. Membranes were then incubated overnight at 4 °C with primary antibodies. After the incubation with primary antibodies, the PVDF membrane was washed with TBST and subsequently incubated for 1 h at room temperature with the corresponding HRP-conjugated secondary antibody. The detection of immune reactive bands was performed by enhanced luminol reagent and visualized by chemiluminescence (1–5 min exposure, Western Lightning Plus-ECL, PerkinElmer, USA, 203-17431). For detecting the phosphorylation of PLN, the PVDF membrane was stripped and re-probed. Densitometric analysis was performed with ImageJ software version 1.52.

### Statistical analysis

Statistical analyses were performed using SPSS 19.0 (IBM Corp., Armonk, USA) and GraphPad Prism 8 (Graphpad Software, USA). Results for the different experimental groups were expressed as mean ± SD. To determine the optimal concentration of CoCl_2_, DFO, TG, Ion and H_2_O_2_ for use in the in vitro cell experiments regression analysis was performed by GraphPad Prism 8 to calculate their *EC*_50_ and *IC*_50_. The differences between groups were analyzed by one-way or two-way analysis of variance (ANOVA) followed by Tukey’s post hoc correction analysis and *χ*^2^ test. We regarded the experimental results (such as microvascular density (MVD), LDH, Mb, CK, protein expression level, etc.) from the left hind limb (non-ischemic side) as the internal control to normalize the levels of relative results of every mouse. Throughout the analysis, *p*-values < 0.05 were considered statistically significant.

## Results

### ABCB5^+^ MSCs alleviate the overloading of Cyto Ca^2+^ in hypoxic HUVECs

**Fig. 1:**
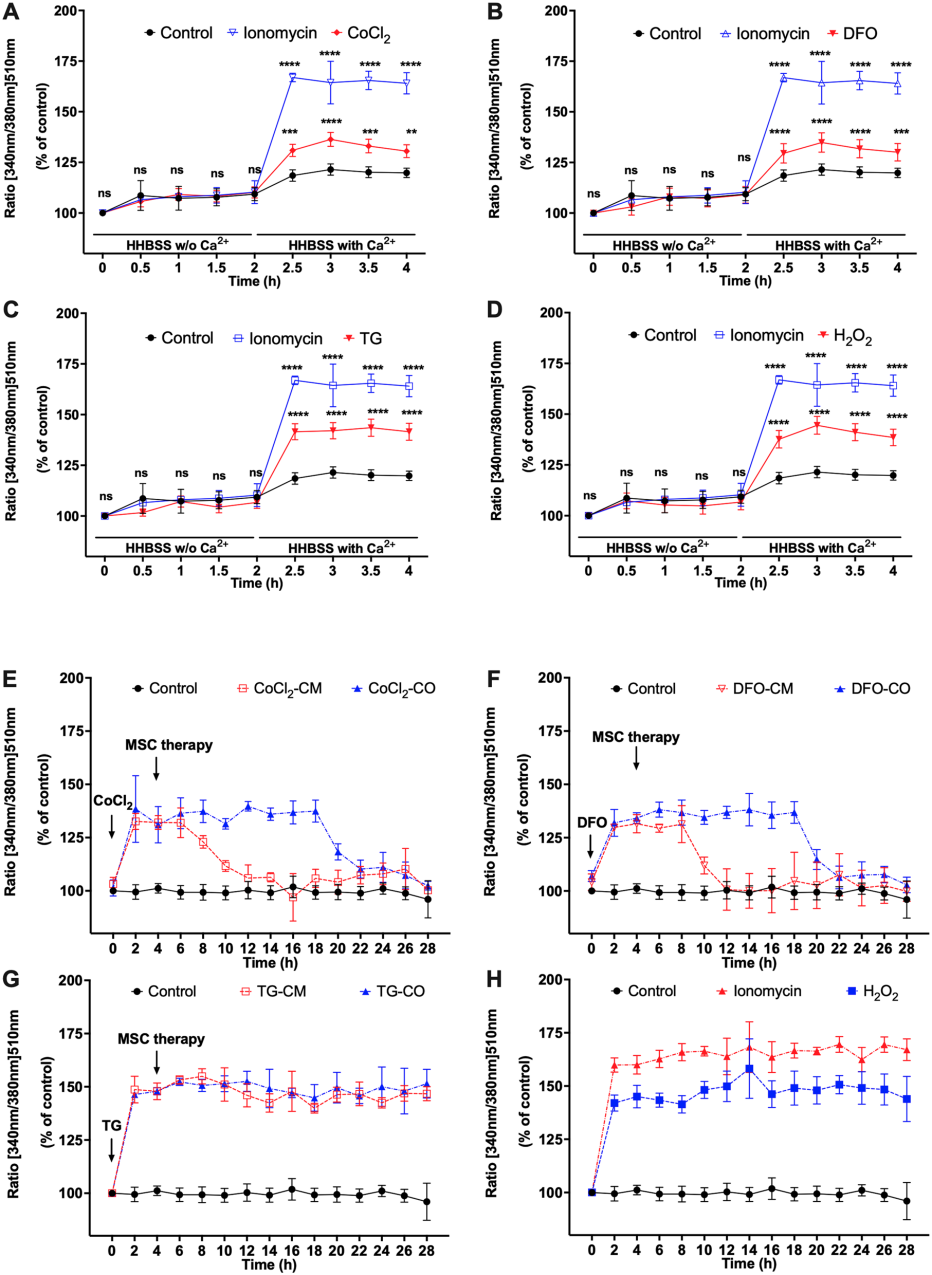
ABCB5^+^ MSCs alleviate overloading of Cyto Ca^2+^ in hypoxic HUVECs. **A**–**D** HUVECs loaded with 2 μM Fura-2-acetoxymethyl ester (Fura 2-AM, Biotium, USA, 50033) were treated with **A** 80 μM CoCl_2_; **B** 120 μM DFO; **C** 3 μM TG; **D** 100 μM H_2_O_2_ in HHBSS buffer without Ca^2+^ for 2 h. After 2 h, buffer was changed to HHBSS (contains DFO, CoCl_2_ or TG) with Ca^2+^. 100 μM Ionomycin (Ion) was used as positive control. The ratio of the fluorescence-emission intensities at 535 nm (340/380 nm) was measured using a Tecan multimode microplate reader by alternating excitation wavelength between 340 and 380 nm. **E**–**G** HUVECs loaded with 2 μM Fura 2-AM were treated with 80 μM CoCl_2_, or 120 μM DFO or 3 μM TG and the 100 μM Ion in medium for 4 h. Afterward, HUEVCs were either co-cultured with ABCB5^+^MSCs (CoCl_2_-CO; DFO-CO; TG-CO) or cultured in ABCB5^+^ MSC-CM (CoCl_2_-CM; DFO-CM, TG-CM) for 24 h. Changes in Fura-2 ratio in HUVECs under **E** CoCl_2_, CoCl_2_-CM and CoCl_2_-CO treatments; **F** DFO, DFO-CM and DFO-CO treatments; **G** TG, TG-CM and TG-CO treatments. **H** Changes in Fura-2 ratio in HUVECs treated by 100 μM Ion and 100 μM H_2_O_2_. Data is represented as mean ± SD, *n* = 3 in each group, ns, not significant, *p < 0.05, **p < 0.01, ***p < 0.001, ****p < 0.0001

To investigate the relationship between extracellular Ca^2+^ and Cyto Ca^2+^ under hypoxia, SERCA2a inhibition and ROS, HUVECs were first treated by CoCl_2_, DFO, TG, H_2_O_2_ and the non-selective calcium ionophore Ion in HHBSS buffer without Ca^2+^ for 2 h. None of the treatments except for H_2_O_2_ (Fig. 1D, $P_{{{\text{H}}_{{2}} {\text{O}}_{{2}} }}$ = 0.9977)_,_ led to a significant change of the Fura-2 ratio when cells were in HHBSS buffer without Ca^2+^ medium compared to non-treated cells (Fig. 1A, $P_{{{\text{CoCl}}_{{2}} }}$ = 0.9484, Fig. 1B, *P*_DFO_ = 0.7792, Fig. 1C, *P*_TG_ = 0.9752, *P*_Ion_ = 0.9355). In the following 2 h, when the HHBSS buffer with Ca^2+^ was used keeping other conditions same, the Fura-2 ratio significantly increased (Fig. 1A, $P_{{{\text{CoCl}}_{{2}} }}$ = 0.0034, Fig. 1B, *P*_DFO_ = 0.0002, Fig. 1C, *P*_TG_ < 0.0001, Fig. 1D, $P_{{{\text{H}}_{{2}} {\text{O}}_{{2}} }}$ < 0.0001, *P*_Ion_ < 0.0001). This indicate extracellular Ca^2+^ influx leading to an increase in Cyto Ca^2+^ concentration, which is in line with the previous studies [22, 23].

Incubation with CoCl_2_ and DFO in cell culture medium lead to a significant increase in Cyto Ca^2+^ indicated by an increase in Fura-2 ratio as compared to the non-treated control HUVECs after 2 h of treatment (Fig. 1E, $P_{{({\text{CoCl}}_{{2}} \;{\text{vs}}.\;{\text{control}}){2}\,{\text{h}}}}$ < 0.0001, Fig. 1F, *P*_(DFO vs. control)2 h_ < 0.0001). Afterward, the addition of MSC-CM or the introduction of MSC-CO, in the continued presence of CoCl_2_ or DFO, led to a slow decrease in the Fura-2 ratio over the following 24 h resembling the Fura-2 ratio of non-treated cells. Although both treatment modalities—conditioned medium as well as co-culture were successful in restoring Cyto Ca^2+^ level similar to that observed in control HUVECs, the MSC-CM treatment led CoCl_2_ or DFO treated HUVECs to reach this level in 12 and 16 h faster in comparison to MSC-CO (Fig. 1E, $P_{{({\text{CoCl}}_{{2}} \;{\text{vs}}.\;{\text{control}}){16}\,{\text{h}}}}$ = 0.5350, $P_{{({\text{CoCl}}_{{2}} - {\text{CO}}\;{\text{vs}}.\;{\text{control}}){16}\,{\text{h}}}}$ = 0.2418, Fig. 1F, *P*_(DFO-CM vs. control)12 h_ = 0.6651, *P*_(DFO-CO vs. control)12 h_ = 0.2625). Addition of TG, ionomycin as well as H_2_O_2_ also lead to increase in Cyto Ca^2+^ (Fig. 1G, *P*_TG_ < 0.0001, Fig. 1H, $P_{{{\text{H}}_{{2}} {\text{O}}_{{2}} }}$ < 0.0001, *P*_Ion_ < 0.0001). However, an additional MSC-CM or MSC-CO treatment did not change Cyto Ca^2+^ levels in the TG treatment group (Fig. 1G, *P*_TG-CM_ < 0.0001, *P*_TG-CO_ < 0.0001).

### ABCB5^+^ MSCs restore ER Ca^2+^ in hypoxic HUVECs

**Fig. 2:**
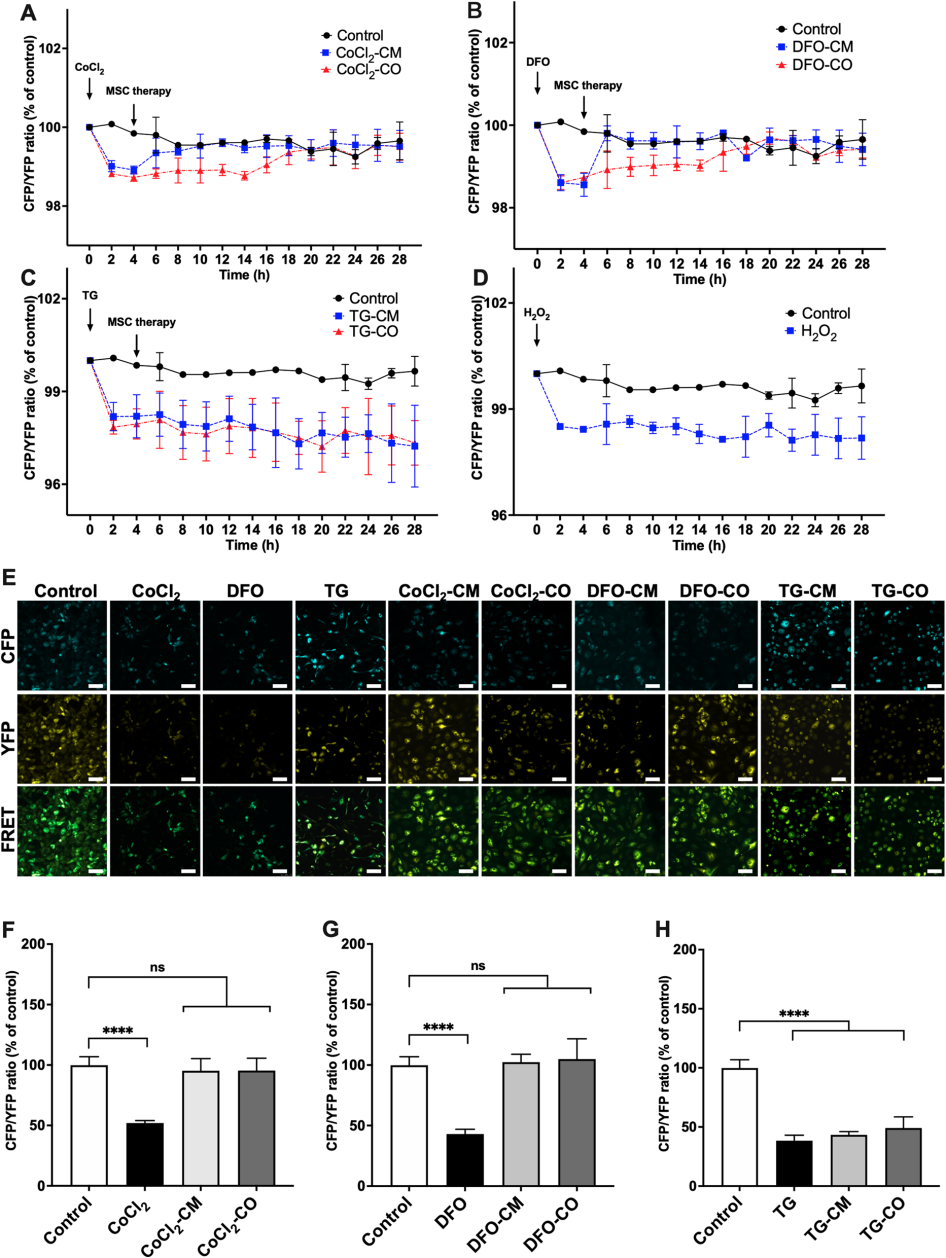
ABCB5^+^ MSCs restore ER Ca^2+^ in hypoxic HUVECs. **A**–**D** HUVECs transduced with the ratiometric ER-Ca^2+^ sensor-D1ER, were treated with 80 μM CoCl_2_, or 120 μM DFO or 3 μM TG for 4 h in medium. Thereafter, HUEVCs were either co-cultured with ABCB5^+^ MSCs (CoCl_2_-CO; DFO-CO; TG-CO) or cultured in ABCB5^+^ MSC-CM (CoCl_2_-CM; DFO-CM; TG-CM) for 24 h. The ratio of CFP (Ex 430 nm/Em 474 nm) intensity by YFP (Ex 514 nm/Em 527 nm) intensity represented the dynamic changes in ER Ca^2+^ concentration and were measured using a Tecan multimode plate reader at every two hours. 100 μM of H_2_O_2_ was used as positive control. Changes in ER-Ca^2+^ in HUVECs under **A** CoCl_2_, CoCl_2_-CM and CoCl_2_-CO treatments; **B** DFO, DFO-CM and DFO-CO treatments; **C** TG, TG-CM and TG-CO treatments; and **D** H_2_O_2_ treatments. **E** Representative images of HUVECs under various treatments taken by SP5 microscopy system (scale bar is 200 μm). **F**–**H** D1ER expressing HUVECs were grown on 13 mm Ø glass coverslips treated with 80 μM CoCl_2_, or 120 μM DFO or 3 μM TG in medium for 4 h. Subsequently, HUEVCs were either co-cultured with ABCB5^+^ MSCs (CoCl_2_-CO; DFO-CO; TG-CO) or cultured in ABCB5^+^ MSC-CM (CoCl_2_-CM; DFO-CM; TG-CM) for 24 h and fixed with 4% PFA. Changes in ER-Ca^2+^ in HUVECs under **F** CoCl_2_, CoCl_2_-CM and CoCl_2_-CO treatments; **G** DFO, DFO-CM and DFO-CO treatments; **H** TG, TG-CM and TG-CO treatments. Data is represented as mean ± SD, *n* = 3 in each group, ns, not significant, *p < 0.05, **p < 0.01, ***p < 0.001, ****p < 0.0001

To detect ER Ca^2+^ change in living HUVECs, the cells were transduced with the D1ER FRET sensor. After 4 h incubation with CoCl_2_, DFO and TG, the FRET ratio in the all conditions significantly decreased compared to the non-treated control HUVECs (Fig. 2A, $P_{{{\text{CoCl}}_{2} }}$ < 0.0001, Fig. 2B, *P*_DFO_ < 0.0001, Fig. 2C, *P*_TG_ = 0.0017, Fig. 2D, $P_{{{\text{H}}_{{2}} {\text{O}}_{{2}} }}$ < 0.0001). The decrease in ER Ca^2+^ induced by CoCl_2_ and DFO treatment was reversed by MSC-CM and MSC-CO treatment over the following 24 h (Fig. 2A, $P_{{({\text{CoCl}}_{{2}} {\text{-CM}}\;{\text{vs}}.\;{\text{control}}){10}\,{\text{h}}}}$ = 0.9877, $P_{{({\text{CoCl}}_{{2}} {\text{-CO}}\;{\text{vs}}.\;{\text{control}}){20}\,{\text{h}}}}$ = 0.9409, Fig. 2B, *P*_(DFO-CM vs. control)6 h_ > 0.9999, *P*_DFO-CO vs. control)20 h_ = 0.1848). Again, MSC-CM led to an earlier restoration of ER Ca^2+^ reversal than MSC-CO. After incubation with TG, the FRET ratio decreased (Fig. 2C, *P*_(TG vs. control)2 h_ = 0.0002) and could not be reversed by MSC-CM or MSC-CO (Fig. 2C, *P*_(TG-CM vs. control)20 h_ < 0.0045, *P*_(TG-CO vs. control)20 h_ = 0.0003). H_2_O_2_ treatment also significantly decreased the FRET ratio as compared to the control group (Fig. 2D, $P_{{({\text{H}}_{2} {\text{O}}_{{2}} \;{\text{vs}}.\;{\text{control}}){2}\,{\text{h}}}}$ < 0.0001).

We also performed acceptor photo bleaching using fixed cell samples (Fig. 2E). Similar to the live cell experiments, here we also found that HUVECs treated with CoCl_2_, DFO and TG displayed a significantly lower FRET ratio than the non-treated controls (Fig. 2F, $P_{{{\text{CoCl}}_{2} }}$ < 0.0001, Fig. 2G, *P*_DFO_ < 0.0001, Fig. 2H, *P*_TG_ < 0.0001). In the HUVECs treated with MSC-CM and MSC-CO treatment, the FRET ratio normalized back to the base line levels (Fig. 2F, $P_{{{\text{CoCl}}_{2} {\text{-CM}}}}$ = 0.8491, $P_{{{\text{CoCl}}_{2} {\text{-CO}}}}$ = 0.9545, Fig. 2G, *P*_DFO-CM_ = 0.3504, *P*_DFO-CO_ = 0.2102). Again, MSC-CM and MSC-CO treatment were not effective in HUVECs treated with TG (Fig. 2H, *P*_TG-CM_ < 0.0001, *P*_TG-CO_ = 0.0001).

### ***ABCB5***^+^***MSCs alleviated hypoxia-induced ROS burden in HUVECs***

**Fig. 3:**
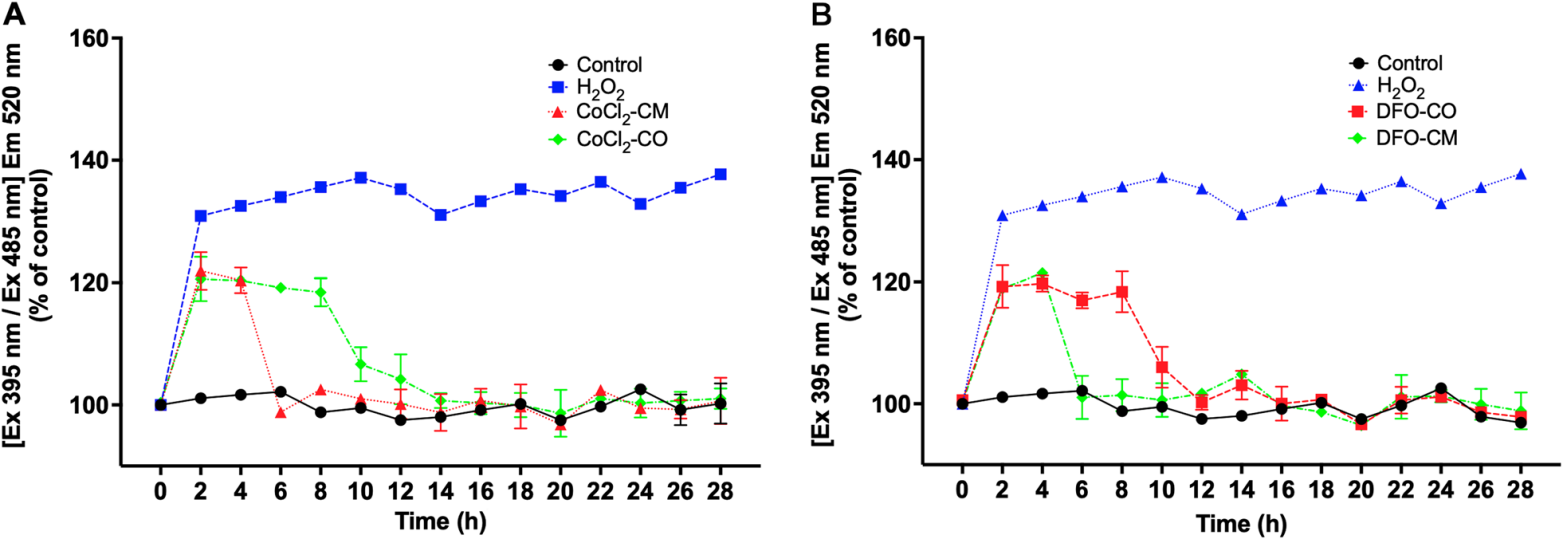
ABCB5^+^ MSCs alleviated hypoxia-induced ROS burden in HUVECs. **A**, **B** HUVECs expressing roGFP3 were treated with 80 μM CoCl_2_, or 120 μM DFO or 100 μM H_2_O_2_ in medium for 4 h. H_2_O_2_ was used as positive control. Thereafter, HUEVCs were either co-cultured with ABCB5^+^ MSCs (CoCl_2_-CO; DFO-CO) or cultured in ABCB5^+^ MSC-CM (CoCl_2_-CM; DFO-CM) for 24 h. The ratio of florescence intensity Ex 395 nm and at Ex 485 nm, and the emission at 520 nm was measured by a Tecan multimode reader every 2 h and ratio was calculated. **A** ROS changes in HUVECs treated by CoCl_2_, CoCl_2_-CM and CoCl_2_-CO treatments; **B** DFO, DFO-CM and DFO-CO treatments. Data is represented as mean ± SD, *n* = 3 in each group

Hypoxia-mediated ROS formation can also interfere with Ca^2+^ homeostasis [49, 50]. Therefore, we next evaluated dynamic changes of ROS. To this end, roGFP3 expressing HUVECs were challenged with CoCl_2_ and DFO. A significant increase in ROS was observed in HUVECs incubated with CoCl_2_ and DFO in the first 4 h (Fig. 3A, $P_{{({\text{CoCl}}_{{2}} \;{\text{vs}}.\;{\text{control}}){4}\,{\text{h}}}}$ < 0.0001, Fig. 3B, *P*_(DFO vs. control)4 h_ < 0.0001, *P*_(TG vs. control)4 h_ = 0.0017). Incubation with H_2_O_2_ (used as control) also led to a similar increase (Fig. 3A, $P_{{{\text{H}}_{{2}} {\text{O}}_{{2}} }}$ < 0.0001). Additional incubation with MSC-CM and MSC-CO reversed this increase in both CoCl_2_ and DFO groups in the following 24 h (Fig. 3A, $P_{{({\text{CoCl}}_{{2}} {\text{-CM}}\;{\text{vs}}.\;{\text{control}}){24}\,{\text{h}}}}$ = 0.9791, $P_{{({\text{CoCl}}_{{2}} {\text{-CO}}\;{\text{vs}}.\;{\text{control}}){24}\,{\text{h}}}}$ = 0.8847, Fig. 3B, *P*_(DFO-CM vs. control)24 h_ = 0.2295, *P*_(DFO-CO vs. control)24 h_ = 0.7574). Here as well, MSC-CM was faster than MSC-CO to bring the ROS to basal level.

### In-vitro ABCB5^+^ MSCs restore ER Ca^2+^ using the SERCA2a-PLN axis

**Fig. 4:**
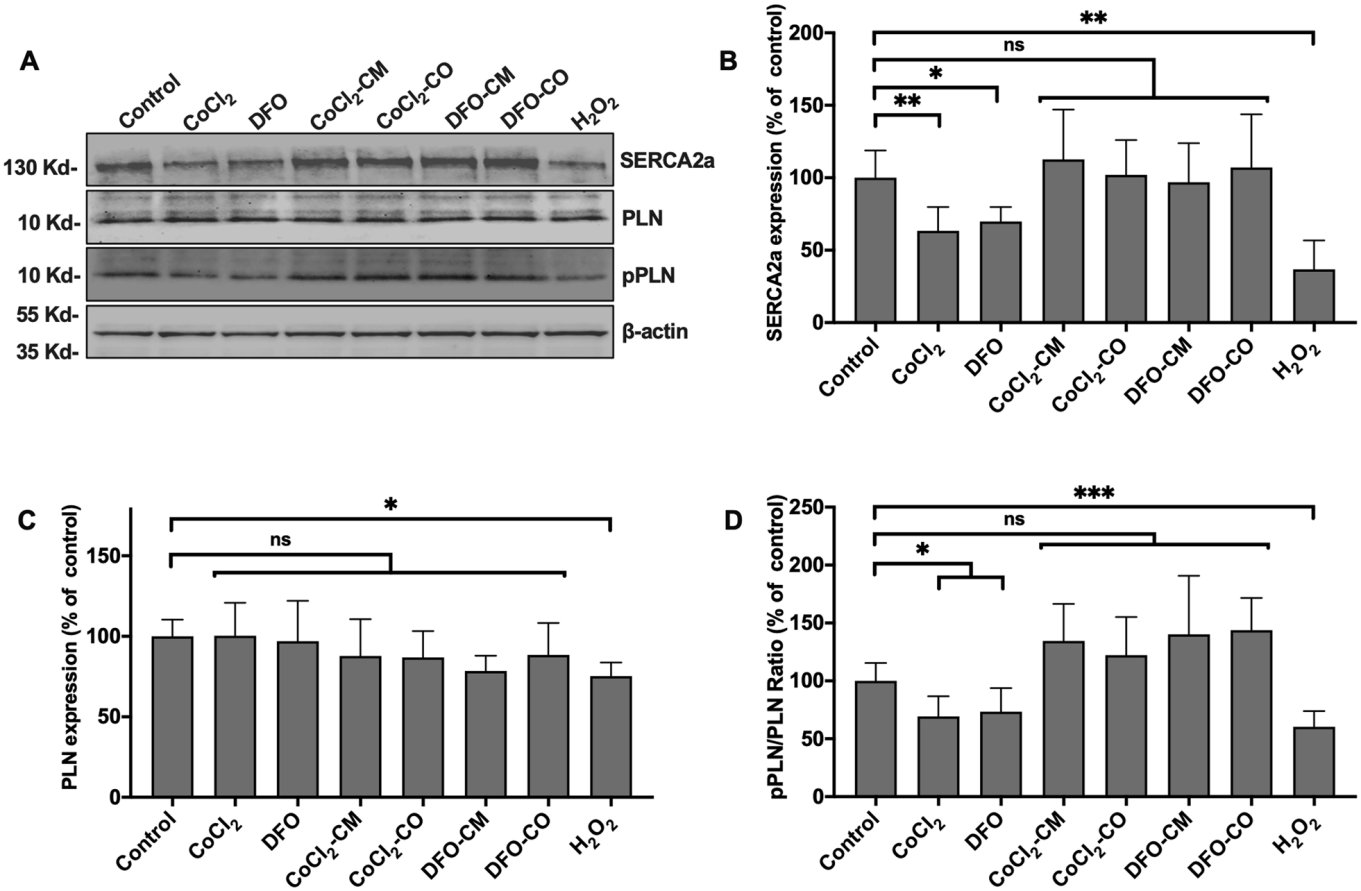
In-vitro ABCB5^+^ MSCs restore ER Ca^2+^ using the SERCA2a-PLN axis. **A** The representative western blot showing SERCA2a, PLN, pPLN and β-actin expression in HUVECs under different treatments. **B**–**D** HUVECs were treated with 80 μM CoCl_2_, or 120 μM DFO or 100 μM H_2_O_2_ for duration of 4 h. Afterward, HUEVCs were either co-cultured with ABCB5^+^ MSCs (CoCl_2_-CO; DFO-CO) or cultured in ABCB5^+^ MSC-CM (CoCl_2_-CM; DFO-CM) for 24 h. The expression of SERCA2a, PLN, Phosphorylated PLN (pPLN) and β-actin expression was determined by western blotting and quantified by densitometry using NIH ImageJ 1.52 software. Expression of SERCA2a and PLN is presented as fold change compared to untreated control cells while pPLN is shown as ratio of pPLN/PLN. Quantitative analysis of **B** SERCA2a, (normalized to β-actin) and **C** PLN expression (normalized to β-actin); and **D** ratio pPLN to PLN

Hypoxia and expression of HIF-1α has been implicated in the suppression of SERCA2a expression [51]. Therefore, we assessed if this was also case in hypoxic HUVECs. WB results showed that incubation over 4 h with CoCl_2_, DFO and H_2_O_2_ led to a decrease in SERCA2a expression as compared to the control group (Fig. 4B, $P_{{{\text{CoCl}}_{2} }}$ = 0.0042, *P*_DFO_ = 0.0153, $P_{{{\text{H}}_{{2}} {\text{O}}_{{2}} }}$ = 0.0026). When cells were incubated with MSC-CM and MSC-CO in addition to CoCl_2,_ SERCA2a expression increased to a similar level as the control group (Fig. 4B, $P_{{{\text{CoCl}}_{2} {\text{-CM}}}}$ = 0.9193, $P_{{{\text{CoCl}}_{2} {\text{-CO}}}}$ = 0.9997, *P*_DFO-CM_ = 0.9989, *P*_DFO-CO_ = 0.9914). The phosphorylation of PLN decreased significantly after incubation with CoCl_2_, DFO and H_2_O_2_ (Fig. 4D, $P_{{{\text{CoCl}}_{2} }}$ = 0.0255, *P*_DFO_ = 0.0287, $P_{{{\text{H}}_{{2}} {\text{O}}_{{2}} }}$ = 0.0005). However, the ratio of pPLN/PLN under exposure of the cells with MSC-CM and MSC-CO did not change significantly as compared to the control group (Fig. 4D, $P_{{{\text{CoCl}}_{2} {\text{-CM}}}}$ = 0.1827, $P_{{{\text{CoCl}}_{2} {\text{-CO}}}}$ = 0.7414, *P*_DFO-CM_ = 0.5438, *P*_DFO-CO_ = 0.1840).

### ***ABCB5***^+^***MSCs restore the angiogenic function of hypoxic HUVECs***

**Fig. 5:**
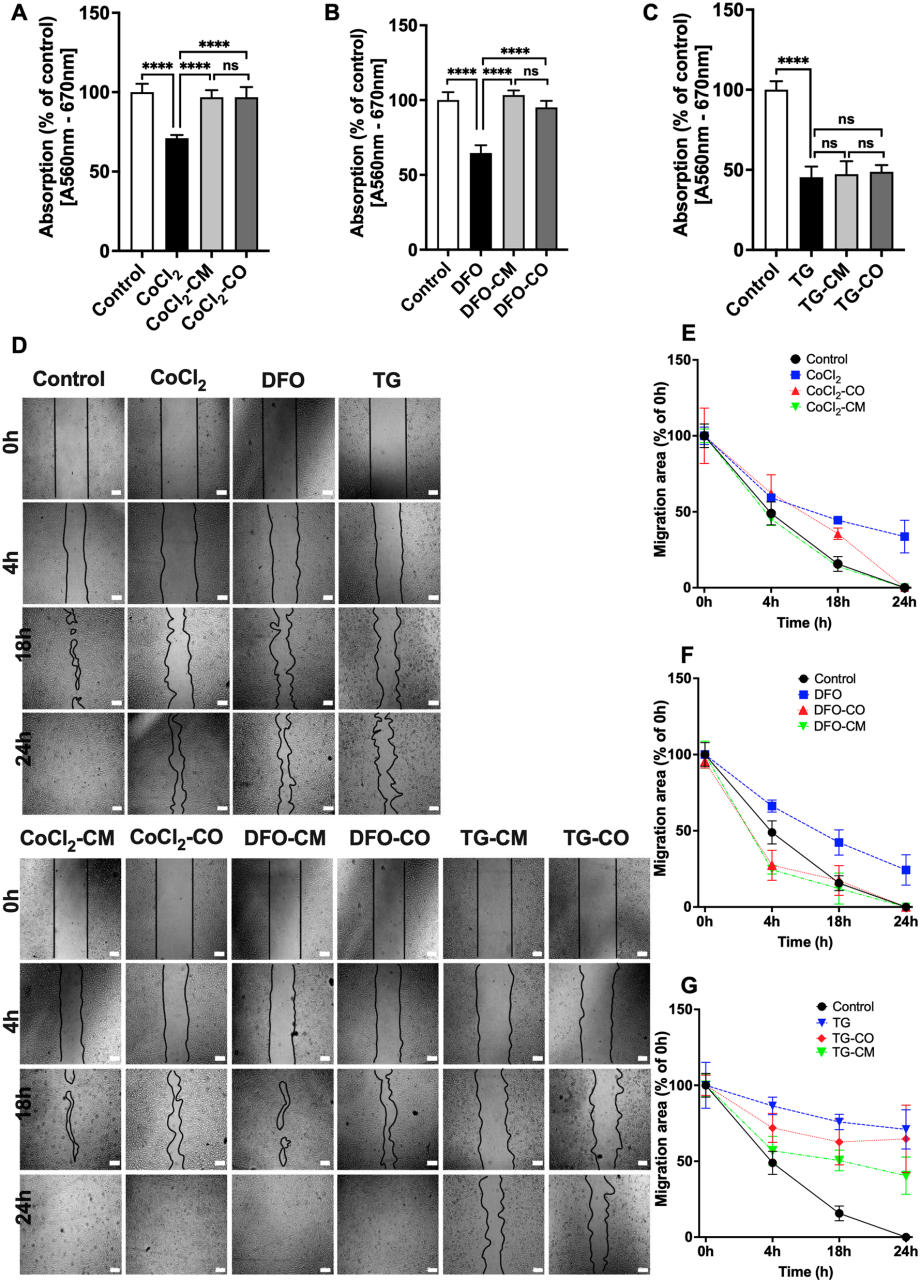
ABCB5^+^ conditioned medium and co-culture both rescue metabolic activity and migration properties of hypoxic HUVECs. **A**–**C** HUVECs were treated either with 80 μM CoCl_2_ or 120 μM DFO for 4 h to induce hypoxia. HUVECs were also treated with 3 μM of TG. Afterward, HUVECs were either co-cultured with ABCB5^+^ MSCs (CoCl_2_-CO; DFO-CO; TG-CO) or cultured in ABCB5^+^ MSC conditioned medium (CoCl_2_-CM; DFO-CM; TG-CM) for 24 h. HUVECs without treatment served as control. Cellular metabolic activity of HUVECs was measured by MTT assay which is an indirect indicator of cell viability. HUVECs metabolic activity under **A** CoCl_2_, CoCl_2_-CM and CoCl_2_-CO treatments; **B** DFO, DFO-CM and DFO-CO treatments; **C** TG, TG-CM and TG-CO treatments. **D** Representative figure showing HUVECs migration under different treatments (scale bar is 200 μm). **E**–**G** Scratch assays were used to study migratory properties of HUVECs. After treatment with 80 μM CoCl_2_ or 120 μM DFO or 3 μM TG for 4 h, HUVECs monolayer was scratched along a straight line. Afterward, HUVECs were either co-cultured with the ABCB5^+^ cells (CoCl_2_-CO; DFO-CO; TG-CO) or cultured with ABCB5^+^ conditioned medium (CoCl_2_-CM; DFO-CM; TG-CM). The photos of the monolayer were taken at 0 h, 6 h, 12 h, and 24 h by an inverted microscope (Axiovert 200 M; Zeiss, Jena, Germany). The gap area was quantitatively evaluated using NIH ImageJ software (version 1.52, Bethesda, USA). Migration rate of HUVECs under **E** CoCl_2_, CoCl_2_-CM and CoCl_2_-CO treatments; **F** DFO, DFO-CM and DFO-CO treatments; **G** TG, TG-CM and TG-CO treatments. Data is represented as mean ± SD, *n* = 3 in each group, ns, not significant, *p < 0.05, **p < 0.01, ***p < 0.001, ****p < 0.0001

We evaluated the metabolic activity of HUVECs under chemically induced hypoxia upon treatment with CoCl_2_ and DFO alone or in combination with MSC-CM and MSC-CO. CoCl_2_ and DFO treatments significantly decreased HUVECs metabolic activity compared to controls (Fig. 5A, $P_{{{\text{CoCl}}_{2} }}$ < 0.0001, Fig. 5B, *P*_DFO_ < 0.0001). However, the MSC-CM and MSC-CO could counter the decrease in HUVECs metabolic activity (Fig. 5A, $P_{{{\text{CoCl}}_{2} {\text{-CM}}}}$ = 0.3758, $P_{{{\text{CoCl}}_{2} {\text{-CO}}}}$ = 0.3666, Fig. 5B, *P*_DFO-CM_ = 0.3005, *P*_DFO-CO_ = 0.0766). To prove the influence of ER Ca^2+^ store-depletion on the metabolic activity of HUVECs, the cells were also exposed to the SERCA inhibitor TG. Treatment of HUVECs with TG led to a further decrease in HUVECs metabolic activity (Fig. 5C, *P*_TG_ < 0.0001). Neither conditioned medium nor co-culture with ABCB5^+^ MSCs were able to rescue HUVECs treated with TG (Fig. 5C, *P*_TG-CM_ < 0.0001, *P*_TG-CO_ < 0.0001).

Next, we evaluated if hypoxia affected HUVECs migratory properties using scratch assays (Fig. 5D). Indeed, the cell migration rate was reduced upon CoCl_2_ or DFO treatment while incubation with TG led to an even more pronounced decrease in migration (Fig. 5E, $P_{{{\text{CoCl}}_{2} }}$ < 0.0001, Fig. 5F, *P*_DFO_ = 0.0006, Fig. 5G, *P*_TG_ < 0.0001) compared with untreated HUVECs. Again, MSC-CM and MSC-CO were able to rescue the migration rate in HUVECs under hypoxia except when the treatments were combined with TG (Fig. 5E, $P_{{{\text{CoCl}}_{2} {\text{-CM}}}}$ > 0.9999, $P_{{{\text{CoCl}}_{2} {\text{-CO}}}}$ > 0.9999, Fig. 5F, *P*_DFO-CM_ > 0.9999, *P*_DFO-CO_ > 0.9999, Fig. 5G, *P*_TG-CM_ < 0.0001, *P*_TG-CO_ < 0.0001).

**Fig. 6:**
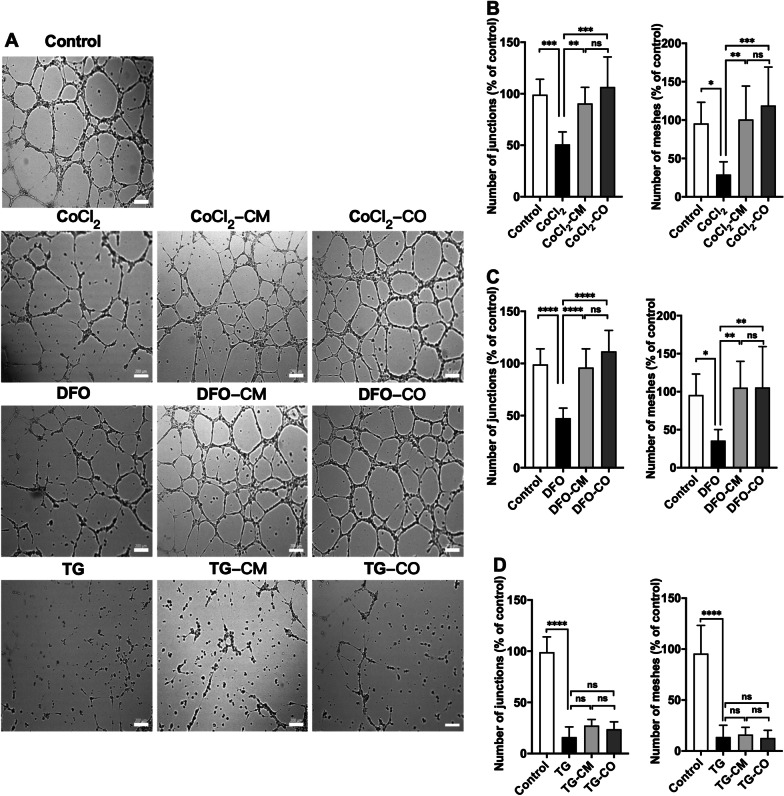
ABCB5^+^ conditioned medium as well as co-culture rescue angiogenic properties of hypoxic HUVECs. **A** Representative images showing tube formation in HUVECs under different culture conditions (scale bar is 200 μm). **B**–**D** HUVECs were treated with 80 μM CoCl_2_ or 120 μM DFO or 3 μM TG for 4 h. Then the HUVECs were harvested and seeded onto the growth factor reduced matrigel pre-coated 24-well plates (2.5 × 10^4^ cells/well). Afterward HUVECs were either co-cultured with ABCB5^+^ MSCs (CoCl_2_-CO; DFO-CO; TG-CO) or cultured in ABCB5^+^ MSC conditioned medium (CoCl_2_-CM; DFO-CM; TG-CM). Images were taken by an inverted microscope (Axiovert 200 M; Zeiss, Jena, Germany) after 6 h of incubation. The number of HUVECs meshes and junctions were analyzed by the angiogenesis analyzer plugin for NIH ImageJ version 1.52. Meshes and junctions after incubation with **B** CoCl_2_, CoCl_2_-CM and CoCl_2_-CO treatments; **C** DFO, DFO-CM and DFO-CO treatments; **D** TG, TG-CM and TG-CO treatments. Data is represented as mean ± SD, *n* = 3 in each group, ns not significant, *p < 0.05, **p < 0.01, ***p < 0.001, ****p < 0.0001

In the tube formation assays, HUVECs displayed the ability to form junctions and meshes automatically under normoxia (Fig. 6A). Under CoCl_2_ and DFO treatment, the number of meshes and junctions significantly reduced compared to untreated HUVECs (Meshes: Fig. 6B, $P_{{{\text{CoCl}}_{2} }}$ = 0.0124, Fig. 6C, *P*_DFO_ = 0.0205, Junctions: Fig. 6B, $P_{{{\text{CoCl}}_{2} }}$ = 0.0003, Fig. 6C, *P*_DFO_ < 0.0001). Incubation with MSC-CM or MSC-CO counteracted this effect (Meshes: Fig. 6B, $P_{{{\text{CoCl}}_{2} {\text{-CM}}}}$ = 0.9939, $P_{{{\text{CoCl}}_{2} {\text{-CO}}}}$ = 0.6444, Fig. 6C, *P*_DFO-CM_ = 0.9566, *P*_DFO-CO_ = 0.9538, Junctions: Fig. 6B, $P_{{{\text{CoCl}}_{2} {\text{-CM}}}}$ = 0.8115, $P_{{{\text{CoCl}}_{2} {\text{-CO}}}}$ = 0.8898, Fig. 6C, *P*_DFO-CM_ = 0.9854, *P*_DFO-CO_ = 0.4850). In the TG treated HUVECs, only few meshes and junctions were formed and neither MSC-CM nor MSC-CO restored the counteracted this effect (Fig. 6D, Meshes: *P*_TG_ < 0.0001, *P*_TG-CM_ < 0.0001, *P*_TG-CO_ < 0.0001, Junctions: *P*_TG_ < 0.0001, *P*_TG-CM_ < 0.0001, *P*_TG-CO_ < 0.0001).

### ABCB5^+^ MSCs decreased weight loss of ApoE^−/−^ mice

**Fig. 7:**
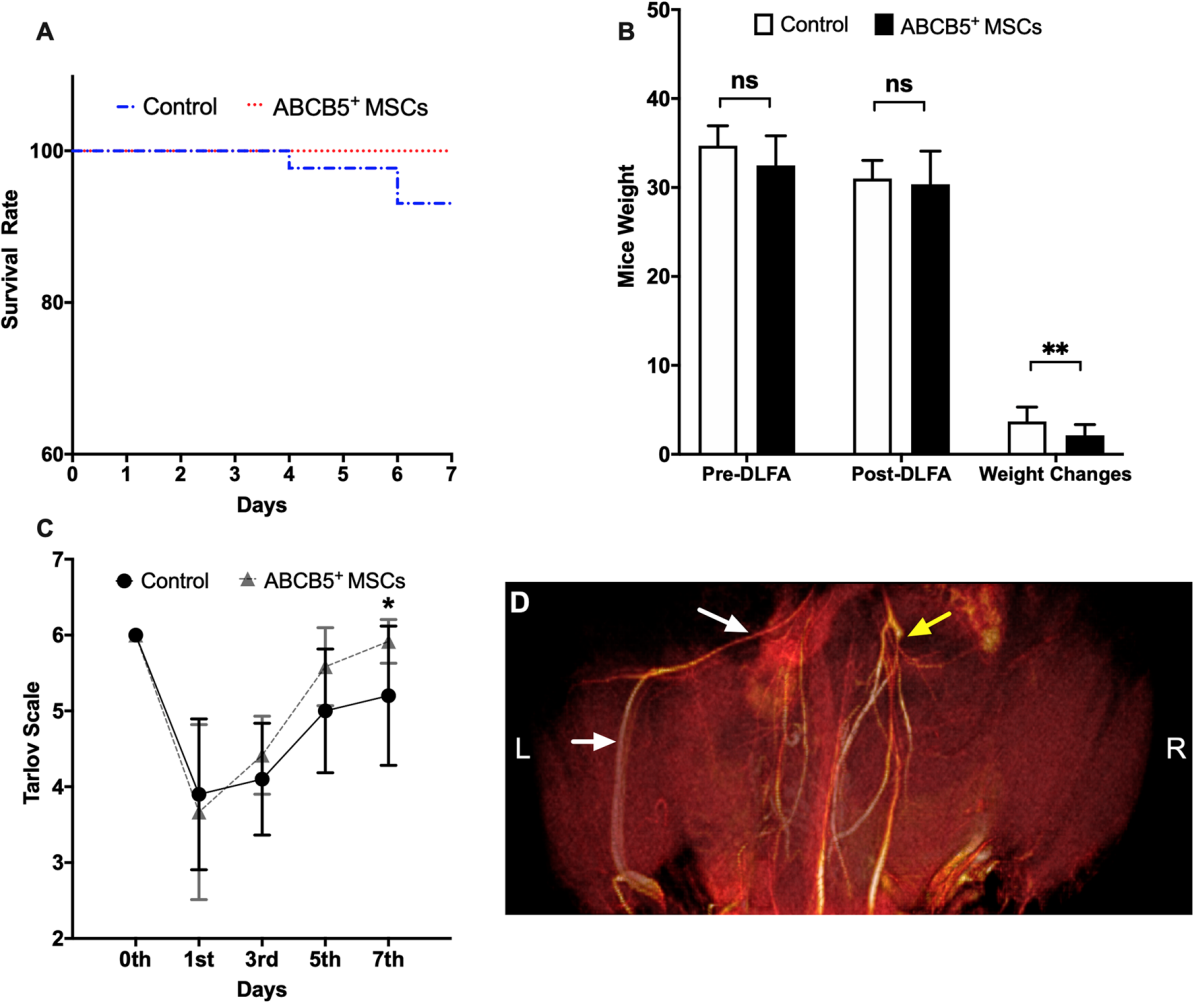
ABCB5^+^ cells therapy in ApoE^−/−^ mice after DLFA leads to reduced weight loss. **A** The survival rate of ApoE^−/−^ mice after DLFA and receiving ABCB5^+^ MSCs (ABCB5^+^ MSCs) or saline (control) treatment (*n* = 12 in each group). **B** Body weight of each of the animals was assessed directly prior to induction of DLFA and 7 days thereafter. **C** Tarlov Scale changes before and 7 days after induction of DLFA. **D** A representative MRI scan after the DLFA operation (scale bar: 5 mm). The right femoral artery (FA) was blocked (yellow arrow) as compared to the left FA (white arrow). Data were represented as mean ± SD, *n* = 12 in ABCB5^+^ MSCs group, *n* = 12 in controls group, ns, not significant, *p < 0.05, **p < 0.01, ***p < 0.001

Double ligation of the femoral artery was performed to induce acute-on-chronic hypoxia in ApoE^−/−^ mice that had been fed on a Western diet for 12 weeks. All 24 mice were successfully operated on. One day after the DLFA, MRI confirmed proximal and distal parts of the right FA were occluded (Fig. 7D). Although, two mice died in the control group on the 4th and 6th days after the procedure yet there was no significant difference in the survival rate of the control group and ABCB5^+^ MSCs treated group (Fig. 7A, *P* = 0.1351). HE sections of the aorta showed atherosclerotic lesions (Additional file 4: Fig. S4A, B) and no differences were observed in the manifestation of aortic AS between both groups (Additional file 4: Fig. S4C, *P* = 0.4652). A comparison of cholesterol and triglycerides in the plasma of the two groups showed no significant differences (Additional file 4: Fig. S4D, *P*_Cholesterol_ = 0.9176, Additional file 4: Fig. S4E, *P*_Triglycerides_ = 0.8828).

To understand the well-being of the mice changes in body weight were observed. Mice were weighed before the DLFA operation (Pre-DLFA) and 7 days after the DLFA operation (Post-DLFA). The mean Pre-DLFA weights of control and ABCB5^+^ MSCs groups were 34.5 ± 2.5 g and 32.8 ± 3.1 g, respectively. There was no significant difference in the Pre-DLFA weights between the two groups (Fig. 7B, *P*_Pre-DLFA_ = 0.1295). The mean weights of control and ABCB5^+^ MSCs groups 7-day Post-DLFA were 30.77 ± 2.15 g and 30.67 ± 3.73 g, respectively. The comparison of 7-day Post-DLFA between the two groups also displayed no significant difference (Fig. 7B, *P*_Post-DLFA_ = 0.9349). However, the ABCB5^+^ MSCs group showed proportionally a significantly lower weight loss than the control group (Fig. 7B, *P* = 0.0096).

### ABCB5^+^ MSCs improved functional recovery of ApoE^−/−^ mice after hind limb ischemia

The motoric function of mice was determined after DLFA using Tarlov Scale. The scores decreased significantly on the 1st day after the DLFA operation in both groups and then increased within the following days. The average functional recovery was similar in the ABCB5^+^ MSCs treated and the control group on the 3rd and the 5th day. However, ABCB5^+^ MSCs treated mice display better motor functions compared to the saline-treated control mice on the 7th day (Fig. 7C, *P* = 0.0185). We did not observe any necrosis or gangrenous tissue after DLFA on the hind limb in any of the mice.

### ABCB5^+^ MSCs therapy improved MVD in ApoE^−/−^ mice after hind limb ischemia

**Fig. 8:**
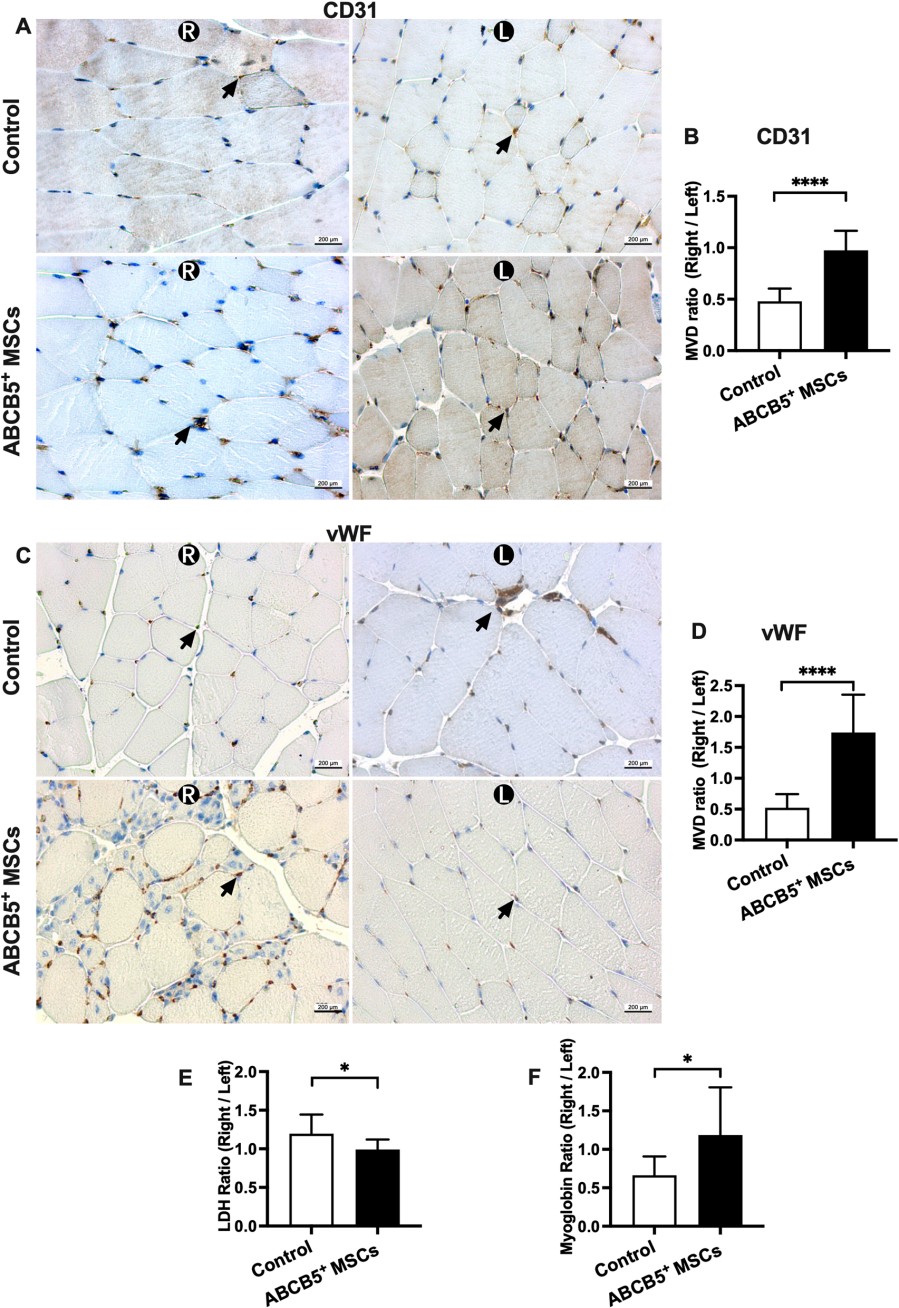
ABCB5^+^ MSCs therapy improve microvascular density in ApoE^−/−^ mice after DLFA. **A**, **C** Representative pictures of IHC staining for **A** CD31 and **B** vWF (black arrow) in bilateral gastronemicus muscles (GM) of the ABCB5^+^ MSCs treated as well as control mice (scale bar: 200 μm). **B**, **D** Comparison of the ratio of **B** CD31 and **D** vWF positive areas in GM sections of ischemic and non-ischemic hindlimbs between control and ABCB5^+^ MSCs groups. **E** LDH ratios levels in VL tissues derived from controls and the ABCB5^+^ MSCs group; **F** Mb ratios level in VL tissues derived from both groups. Data were represented as mean ± SD, *n* = 10 in ABCB5^+^ MSCs group, *n* = 12 in controls group, ns, not significant, *p < 0.05, **p < 0.01, ***p < 0.001, ****p < 0.0001

The ratio of CD31 positive areas in GM sections of ischemic and non-ischemic hind limbs was significantly higher in the ABCB5^+^ MSCs group as compared to the control group (Fig. 8A, B, *P* < 0.0001). As another kind of marker of ECs, the ratio of vWF positive areas in the ABCB5^+^ MSCs group was also significantly higher than in the control group (Fig. 8C, D, *P* < 0.0001). These results indicated that MVD in the ABCB5^+^ MSCs group was higher than in the control group and the ABCB5^+^ MSC therapy displayed a peri-angiogenic effect.

### LDH and Mb in the hind limb muscle

The ratio of LDH enzyme levels in VL tissues of ischemic hind limbs and non-ischemic hind limbs was used to determine muscle damage post DLFA between the two groups. In the ABCB5^+^ MSC group, LDH ratios were significantly lower than in the control group (Fig. 8E, *P* = 0.0200). The Mb ratios of the ABCB5^+^ MSC group were significantly higher than those in the control group (Fig. 8F, *P* = 0.0210).

### SERCA2a and pPLN in hind limb muscle samples

**Fig. 9:**
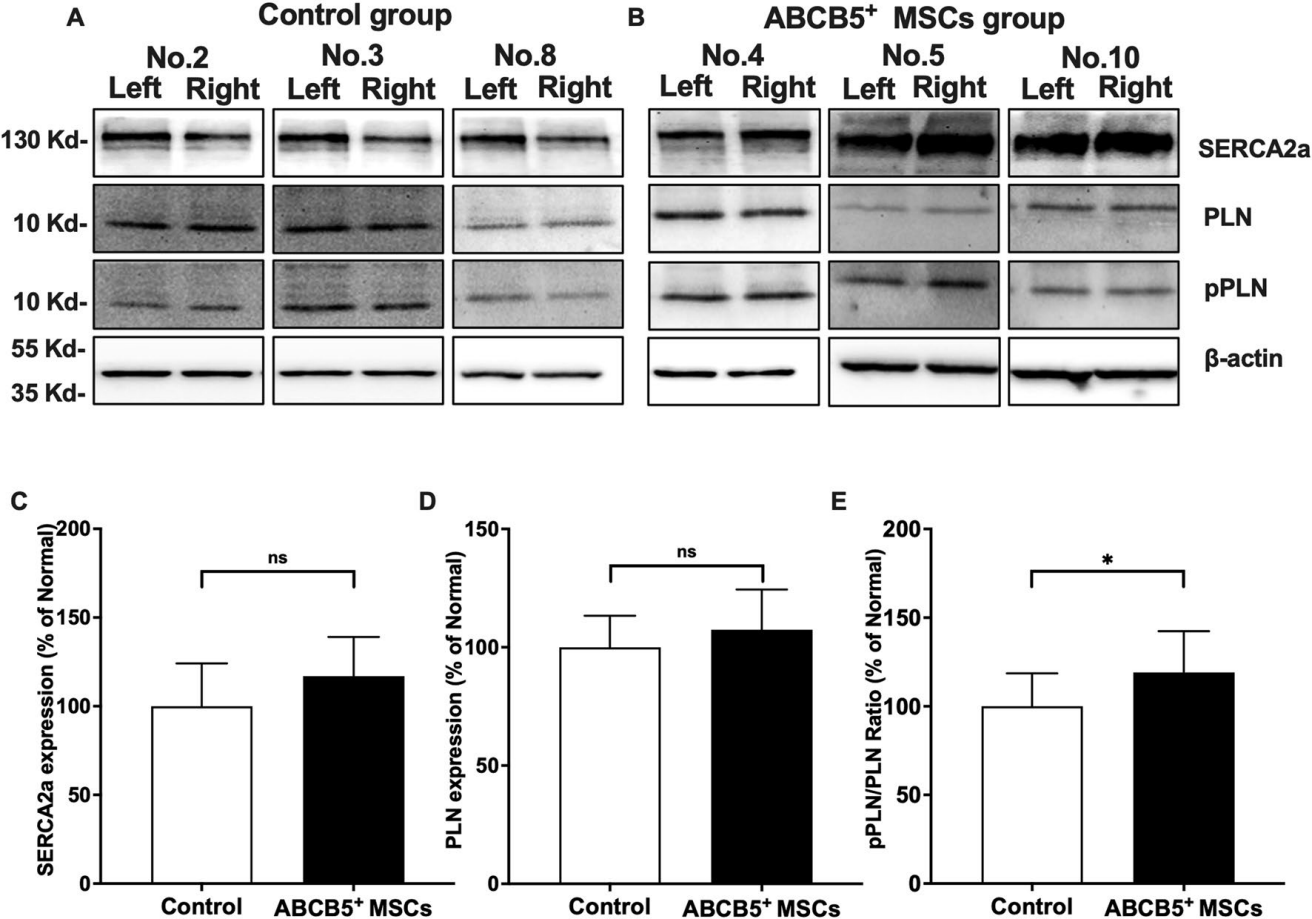
In-vivo ABCB5^+^ MSCs restore ER Ca^2+^ using the SERCA2a-PLN axis. **A**, **B** Representative SERCA2a, PLN, pPLN, and β-actin expression in bilateral gastrocnemius muscle (GM) samples from the 3 mice from control (**A**) and ABCB5 + MSC treated groups (**B**). Full-length blots are presented in Additional file 5: Fig. S5. **C**–**E** Densitometric quantification of **C** SERCA2a expression (normalized to left side), **D** PLN expression (normalized to left side) and **E** pPLN/PLN (normalized to left side). Data were represented as mean ± SD, *n* = 10 control and *n* = 12 in ABCB5^+^ MSCs group, ns, not significant, *p < 0.05

WB was performed to measure expression levels of SERCA2a, PLN and pPLN in GM tissue samples (Fig. 9A, B). The ratio (ischemic/non-ischemic) of SERCA2a expression in ABCB5^+^ MSCs group showed no significant difference (Fig. 9C, *P* = 0.1014). The phosphorylation of PLN in muscle specimens derived from animals of the ABCB5^+^ MSCs group showed a significantly higher ratio (ischemic/non-ischemic) as compared to the control group (Fig. 9E, *P* = 0.0484).

## Discussion

Hypoxia under physiological conditions stimulates angiogenesis [3–5]. However, under pathological circumstances as observed in cerebral ischemia, myocardial infarction and PAD [6, 7], prolonged hypoxia also perturbs Ca^2+^ homeostasis, oxidative stress and cellular apoptosis leading to end organ damage. Although the benefits of some stem cell therapies for PAD have been discussed previously, the influence of ABCB5^+^ MSCs on ECs Ca^2+^ homeostasis (which is essential for angiogenesis [34]) remains to be determined.

In this study, we have demonstrated firstly, in an in-vitro model, that hypoxia leads to Cyto Ca^2+^ increase and ER Ca^2+^ depletion in HUVECs and that MSC-CM or MSC-CO can alleviate this effect. Secondly, we show that MSC-CM or MSC-CO also eliminates hypoxia-mediated ROS stress. Thirdly, we describe that MSC-CM and MSC-CO restore hypoxia perturbed ER-Ca^2+^ via modulation of the expression of SERCA2a and the phosphorylation of PLN. Fourthly, ABCB5^+^ MSCs restore the angiogenic function of hypoxic HUVECs. Finally, we show that ABCB5^+^ MSCs therapy improves MVD and reduced damage to the hind limb muscles in ApoE^−/−^ mice under hind limb ischemia.

As an irreversible inhibitory reagent of SERCA2a, TG could irreversibly bind to the SERCA2a to decrease the ER restoring ability. The results of our research also show that the treatment of TG significantly lead to the ER Ca^2+^ decreasing and Cyto Ca^2+^ increasing. Besides, the experimental results also show that the metabolic activity, migration rate and the tube formation ability of the HUVECs were all significantly decreased under the TG treatment. It demonstrated that the ER Ca^2+^ restoring ability played an important role in the angiogenic function of HUVECs.

Under hypoxia induced by treatment of HUVECs with CoCl_2_ and DFO, an increase in Cyto Ca^2+^ and ER Ca^2+^ liberation was observed. Long-term hypoxia as observed in PAD leads to depletion of cellular ATP. ATP is required by several pumps and ion transporters to maintain a Ca^2+^ gradient not just within various organelles in the cells but also between the cell and the extracellular environment [52]. Dysfunction of these pumps and ion transporters due to ATP exhaustion caused by hypoxia leads to an influx of extracellular Ca^2+^ and store-depletion of ER Ca^2+^. This in turn activates plasma membrane Ca^2+^ channels, leading to an influx of Ca^2+^ into the cytosol resulting in a Cyto Ca^2+^ overload [22, 23, 53] which affects ECs cellular function, as we observed with a significant decrease in metabolic activity, migration rate and reduced number of meshes and junctions in hypoxic HUVECs as compared to the control groups. These perturbations in the normal cellular function of the HUVECs also affect the ECs angiogenic function [54, 55]. Co-culture with ABCB5^+^ MSCs or in ABCB5^+^ MSCs condition medium restored both cytoplasmic as well as ER Ca^2+^ to the pre-hypoxia levels and exerted a restorative effect on metabolic activity and angiogenic function. Although both co-culture and condition medium reversed the effect of hypoxia yet faster recovery was observed in hypoxic HUVECs cultured in ABCB5^+^ MSCs conditioned medium. MSCs secrete different paracrine factors under hypoxia and hyperoxia (21% O_2_) [56], whether ABCB5^+^ MSCs also exhibit such change remains for further investigations.

Treatment of HUVECs with CoCl_2_ and DFO leads to a decrease in the expression of SERCA2a as well as in the PLN phosphorylation rate. This was also observed when HUVECs were treated with only H_2_O_2_, suggesting the role of hypoxia-induced mitochondrial ROS. Indeed, SERCA2a expression has been shown to decrease significantly under hypoxia. This may be induced either by low oxygen concentration [57] or CoCl_2_ treatment [58], which also leads to an increase in ROS. Physiological levels of ROS lead to the formation of glutathione adducts from S-glutathione and SERCA2a in both whole muscle tissue as well as in ECs which maintain the Ca^2+^ store in ER [59, 60]. This is essential for VEGF-induced Ca^2+^ influx and migration which has a direct consequence on angiogenetic signaling [59, 60]. Under pathological conditions. ROS causes excessive oxidation of the cysteine residues in SERCA, resulting in decreased Cyto Ca^2+^ uptake, thus depleting the ER Ca^2+^ stores and causing dysregulated downstream signaling. For instance, SERCA2a activity in myocardium derived from infarction sites is attenuated due to the decrease of phosphorylation of PLN [61–64]. It is already known that accumulated ROS in ECs derails Ca^2+^ homeostasis that potentially resulting in cell death and even end-organ tissue damage [6, 29, 65]. Co-culture with ABCB5^+^ MSCs or culture in ABCB5^+^ MSCs condition medium restored SERCA2a expression and the phosphorylation ratio of PLN in hypoxic HUVECs. This can be attributed to the anti-oxidative effect exerted by MSCs and MSC-CM. Previous studies have reported that the stem cell exerts ROS suppression in a variety of in vitro and in vivo models of disease [66–71]. MSCs and MSC-CM are known to directly scavenge free radicals, bolster endogenous antioxidant defenses [72] and deliver functional mitochondria via extracellular vesicles [73]. This subsequently explains the restorative effect of ABCB5 MSCs and their conditioned medium on SERCA2a expression in hypoxic HUVECs.

Mice receiving ABCB5^+^ MSCs treatment after DLFA exhibited less muscle damage suggested by significantly less release of LDH, and Mb in the GM and VL muscle tissue and improved MVD. Functional recovery after DLFA determined by applying the Tarlov Scale is consistent with the previous reports [74]. Correspondingly to the in vitro WB results, in vivo in the GM muscle lysate, we observed a higher pPLN/PLN ratio in mice receiving ABCB5^+^ MSCs than in the saline-treated control group. However, no difference in the expression level of SERCA2a was observed between ABCB5^+^ MSCs treated mice and controls. The main reason may be that the hind limb not only contains ECs and that other cell types might express a different isoform of SERCA.

We identified some potential limitations in this research. The first limitation is our recent results still could not provide enough evidence to completely elucidate the mechanism of the ABCB5^+^ MSCs therapeutic effect on Ca^2+^ homeostasis of HUVECs under the hypoxia. Maybe the hypoxia-driven changes in the Ca^2+^ homeostasis of HUVECs may also have an effect on the cell biology of the ABCB5^+^ MSCs which could interact again with the HUVECs. So the interaction of ABCB5^+^ MSCs and ECs under hypoxic still needs to be explored. Secondly, because the current assays utilized are only applicable in vitro, the research project didn't mention the Ca^2+^ homeostasis on the ischemic injury. In vivo, we only focused on the protective effect of the ABCB5^+^ MSCs in a model of acute on chronic ischemia. More experiments needed be planned to establish the direct relationship between ABCB5^+^ MSCs, Ca^2+^ homeostasis, and protection from ischemia injury in the future. Finally, although the majority of Cyto Ca^2+^ is eliminated by SERCA2a (74%), Cyto Ca^2+^ is also eliminated via alternative ways such as the Na^+^/Ca^2+^ exchanger (24%), the sarcolemmal Ca^2+^ ATPase (1%) and the mitochondrial uniporter (1%) [75]. It remains uncertain how much of the ER Ca^2+^ restoring ability is mediated by SERCA2a and PLN. Here knock-down experiments using siRNA to silence SERCA2a will be helpful in further research.

## Conclusion

In vitro, we demonstrated that the hypoxia can functionally impair the ER Ca^2+^ restoring ability by inducing downregulation of SERCA2a expression and PLN phosphorylation in HUVECs, which then disrupts the ER Ca^2+^ homeostasis and hereby perturbs the angiogenic function. We further suggest that ROS may play a role in this process. ABCB5^+^ MSCs therapy could restore the homeostasis of ER Ca^2+^ and Cyto Ca^2+^ by increasing SERCA2a expression and the phosphorylation of PLN, hereby enhancing the angiogenic function of ECs under hypoxia in vitro and under hind limb ischemia in vivo. This provides new evidence of a protective effect of ABCB5^+^ MSCs in ischemia and supports the application of ABCB5^+^ MSCs for novel therapeutic approaches in the future.

## Supplementary Information

**Additional file 1: Figure S1**. Dose and kinetics of CoCl_2_. HUVECs were treated with different concentration of CoCl_2_ for different duration of time and HIF-1α expression levels was evaluated via western blotting. **A** A representative WB showing HIF-1α expression levels in HUVECs. HIF-1α increased in a dose-dependent manner after incubation with CoCl_2_ for 4 h. Regression analysis of densitometric quantification yielded an *EC*_50_ of 15.68 μM. **B** A representative WB displaying that HIF-1α expression increased in a time-dependent fashion under 100 μM CoCl_2_ treatment and peaked at 4 h of treatment and stayed steady until 24 h. **C** HUVECs transduced with roGFP3 displayed an increase in ROS signal when subjected to increasing concentration of CoCl_2_. Regression analysis indicated that *EC*_50_ for ROS under CoCl_2_ treatment was 80.33 μM. Response to 100 μM H_2_O_2_ was noted as maximum ROS response (100%) and was used to normalize response obtained upon CoCl_2_ treatment. **D** HUVECs metabolic activity measured via MTT, decreased with increasing CoCl_2_ concentration. An *IC*_50_ of 126.7 μM was determined by regression analysis. Data were represented as mean ± SD, *n* = 3, ns, not significant, *p < 0.05.

**Additional file 2: Figure S2**. Dose and kinetics of DFO. HUVECs were treated with different concentrations of DFO for 4 h and HIF-1α expression levels was evaluated using western blots (WB). **A** A representative WB showing that HIF-1α expression increased in a dose-dependent manner under incubation with DFO and regression analysis indicated an *EC*_50_ of 63.68 μM. **B** HIF-1α expression levels increased time-dependently under 100 μM DFO. Its expression level significantly increased after 2 h and reached a plateau at 4 h of DFO treatment. **C** HUVECs transduced with roGFP3 displayed an increase in ROS signal when subjected to increasing concentration of DFO. Regression analysis revealed the *EC*_50_ to be 121.9 μM. Response to 100 μM H_2_O_2_ was noted as maximum ROS response (100%) and was used to normalize responses obtained for DFO treatment. **D** A decrease in the metabolic activity of HUVECs which corresponded to the increase of DFO concentration was observed in MTT assay. An *IC*_50_ of 169.7 μM was determined by regression analysis. Data were represented as mean ± SD, *n* = 3, ns, not significant, *p < 0.05.

**Additional file 3: Figure S3**. IC_50_ of Thapsigargin (TG), Hydrogen Peroxide (H_2_O_2_), and Ionomycin (Ion). HUVECs were subjected to different concentrations of TG, H_2_O_2_ and Ion for 4 h and MTT assay was performed. **A** HUVECs metabolic activity decreased with the increase of TG concentrations. Regression analysis was performed and *IC*_50_ of TG on HUVECs was found to be 3417 nM. **B** HUVECs metabolic activity also decreased with the increase of H_2_O_2_ concentrations. Regression analysis showed *IC*_50_ of H_2_O_2_ on HUVECs to be 131.3 μM. **C** HUVECs metabolic activity decreased with the increasing concentrations of Ion. Regression analysis yielded the *IC*_50_ of Ion on HUVECs as 117.4 μM. Data were represented as mean ± SD, *n* = 3, ns, not significant, *p < 0.05.

**Additional file 4: Figure S4**. No significant difference was observed in AS lesions, cholesterol, triglycerides between ABCB5^+^ MSC treated and untreated mice. 7 days post DLFA, blood and aorta samples from mice were taken. A representative HE stained sections of the aorta from the **A** control group and **B** ABCB5^+^ MSC treated mice. A1 and B1 displays atherosclerotic vessel wall while A2 and B2 displays normal vessel wall (scale bar is 50 μm in Figure A and B; and 200 μM in Figure A1, A2, B1, B2). **C** Comparison of aortic AS lesion appearance between ABCB5^+^ MSC treated and control mice. **D** Triglyceride concentration and **E** Cholesterol concentration in plasma between two groups of mice. Data were represented as mean ± SD, n = 10 in ABCB5^+^ MSCs group, *n* = 12 in controls group, ns, not significant.

**Additional file 5: Figure S5**. Full-length blots. **A**, **B** Full-length blots of SERCA2a, PLN, pPLN, and β-actin expression in left (L) and right (R) sides from control (A) and ABCB5^+^ MSC treated groups (B).

## Data Availability

The datasets used and analyzed during the current study are available from the corresponding author on reasonable request.

## Abbreviations

PAD: Peripheral artery disease
ECs: Endothelial cells
ABCB5^+^ MSCs: ATP-binding cassette subfamily B member 5 positive mesenchymal stromal cells
VEGF: Vascular endothelial growth factor
ROS: Reactive oxygen species
HIF: Hypoxia-inducible factor
ER: Endoplasmic reticulum
Cyto: Cytosolic
SERCA2a: Sarco-/endoplasmic reticulum ATPase 2a
PLN: Phospholamban
ApoE^−/−^: Apolipoprotein E knock-out
HUVECs: Human umbilical cord vein endothelial cells
vWF: Willebrand factor
FBS: Fetal bovine serum
P/S: Penicillin and streptomycin
CoCl_2_: Cobalt (II) chloride
DFO: Deferoxamine
TG: Thapsigargin
ABCB5^+^ MSC-CO: ATP-binding cassette subfamily B member 5 positive mesenchymal stromal cell coculture
ABCB5^+^ MSC-CM: ATP-binding cassette subfamily B member 5 positive mesenchymal stromal cell conditioned medium
MTT: 3-(4, 5-Dimethyl thiazolyl-2)-2, 5-diphenyltetrazolium bromide
DMSO: Dimethyl sulfoxide
SDS: Sodium dodecyl sulfate
FRET: Fluorescence resonance energy transfer
YFP: Yellow fluorescent protein
CFP: Cyan fluorescent protein
PFA: Paraformaldehyde
Fura 2-AM: Fura-2-acetoxymethyl ester
H_2_O_2_: Hydrogen peroxide
Ion: Ionomycin
S.C.: Subcutaneous injection
DLFA: Double ligation of the right femoral artery
MRI: Magnetic resonance imaging
FA: Femoral artery
VL: Vastus lateralis
GM: Gastrocnemius
HE: Hematoxylin & Eosin
IHC: Immunohistochemistry
AS: Atherosclerosis
LDH: Lactate dehydrogenase
DTT: Dithiothreitol
Mb: Myoglobin
CK: Creatine kinase
WB: Western blot
PVDF: Polyvinylidene fluoride
TBST: Tris-buffered saline with 0.1% Tween 20
ANOVA: Analysis of variance
MVD: Microvascular density
Pre-DLFA: Before the DLFA operation
Post-DLFA: After the DLFA operation
