# Supplementary figures and images for: ABCB5+ mesenchymal stromal cells therapy protects from hypoxia by restoring Ca2+ homeostasis in vitro and in vivo
Source: Stem Cell Res Ther. 2023 Feb 9;14:24. doi: 10.1186/s13287-022-03228-w (PMC9912525; doi:10.1186/s13287-022-03228-w)

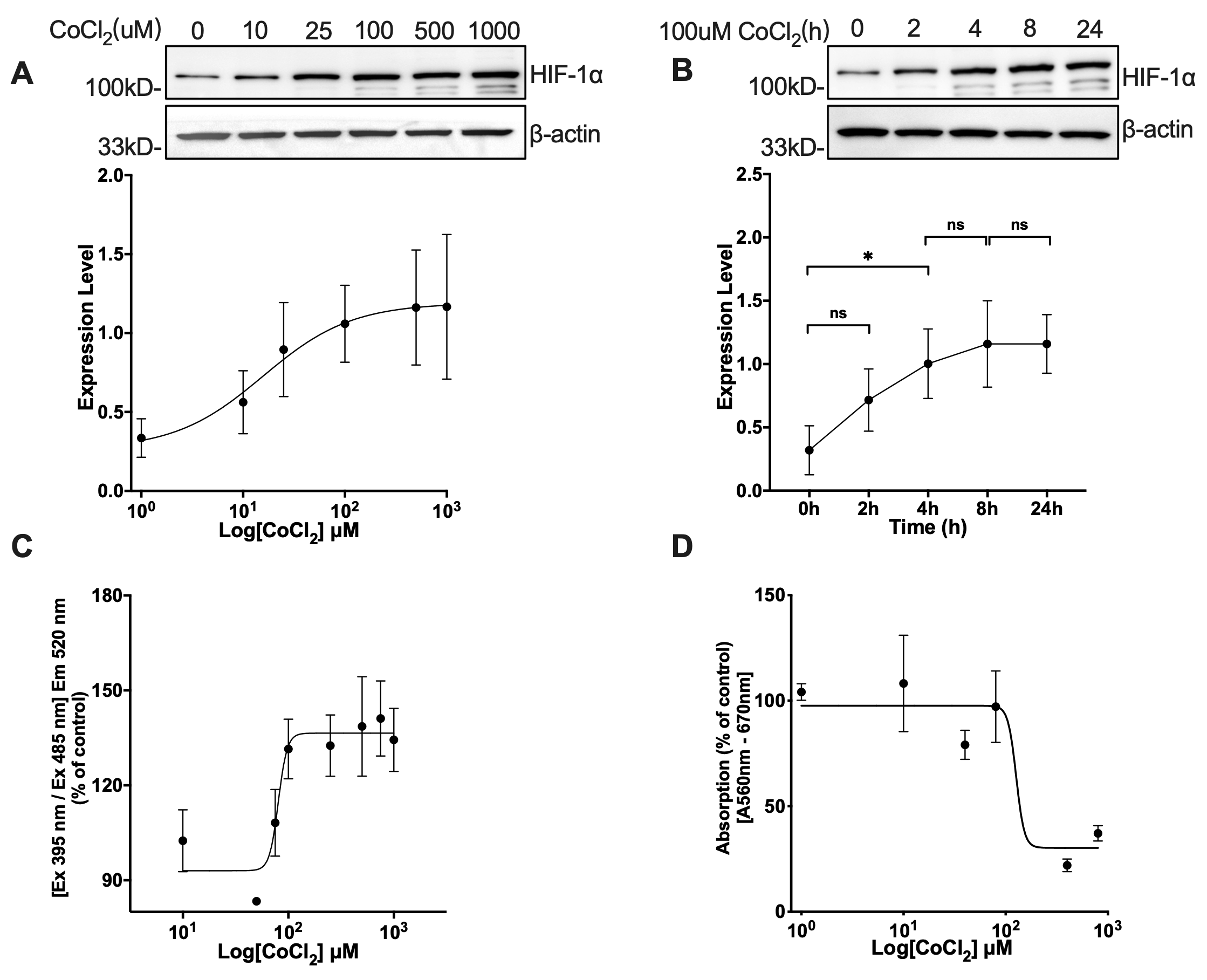

Supplement: Supplementary file 1 — Additional file 1: Figure S1. Dose and kinetics of CoCl2. HUVECs were treated with different concentration of CoCl2 for different duration of time and HIF-1α expression levels was evaluated via western blotting. A A representative WB showing HIF-1α expression levels in HUVECs. HIF-1α increased in a dose-dependent manner after incubation with CoCl2 for 4 h. Regression analysis of densitometric quantification yielded an EC50 of 15.68 μM. B A representative WB displaying that HIF-1α expression increased in a time-dependent fashion under 100 μM CoCl2 treatment and peaked at 4 h of treatment and stayed steady until 24 h. C HUVECs transduced with roGFP3 displayed an increase in ROS signal when subjected to increasing concentration of CoCl2. Regression analysis indicated that EC50 for ROS under CoCl2 treatment was 80.33 μM. Response to 100 μM H2O2 was noted as maximum ROS response (100%) and was used to normalize response obtained upon CoCl2 treatment. D HUVECs metabolic activity measured via MTT, decreased with increasing CoCl2 concentration. An IC50 of 126.7 μM was determined by regression analysis. Data were represented as mean ± SD, n = 3, ns, not significant, *p < 0.05. [file 13287_2022_3228_MOESM1_ESM.tiff]

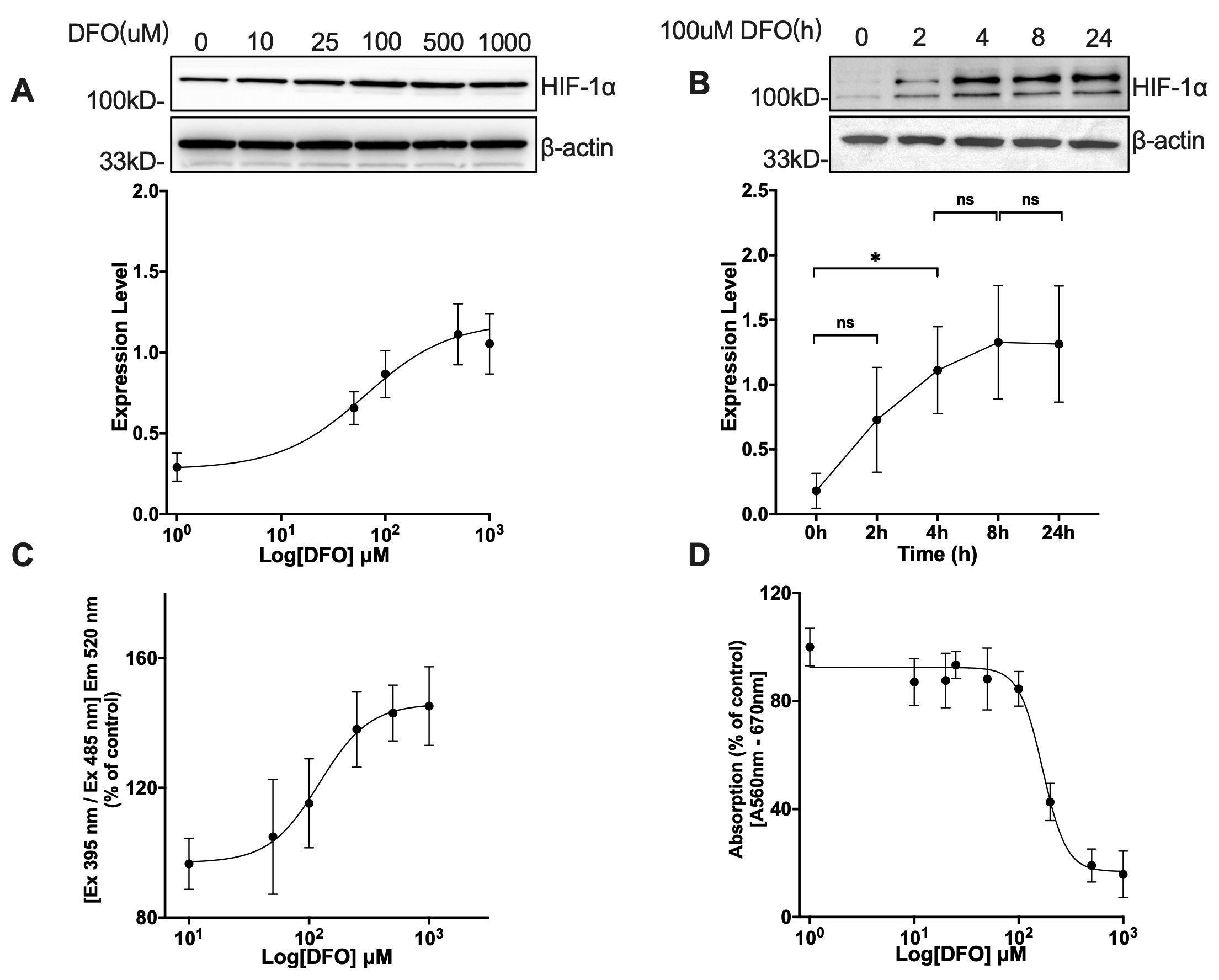

Supplement: Supplementary file 2 — Additional file 2: Figure S2. Dose and kinetics of DFO. HUVECs were treated with different concentrations of DFO for 4 h and HIF-1α expression levels was evaluated using western blots (WB). A A representative WB showing that HIF-1α expression increased in a dose-dependent manner under incubation with DFO and regression analysis indicated an EC50 of 63.68 μM. B HIF-1α expression levels increased time-dependently under 100 μM DFO. Its expression level significantly increased after 2 h and reached a plateau at 4 h of DFO treatment. C HUVECs transduced with roGFP3 displayed an increase in ROS signal when subjected to increasing concentration of DFO. Regression analysis revealed the EC50 to be 121.9 μM. Response to 100 μM H2O2 was noted as maximum ROS response (100%) and was used to normalize responses obtained for DFO treatment. D A decrease in the metabolic activity of HUVECs which corresponded to the increase of DFO concentration was observed in MTT assay. An IC50 of 169.7 μM was determined by regression analysis. Data were represented as mean ± SD, n = 3, ns, not significant, *p < 0.05. [file 13287_2022_3228_MOESM2_ESM.tiff]

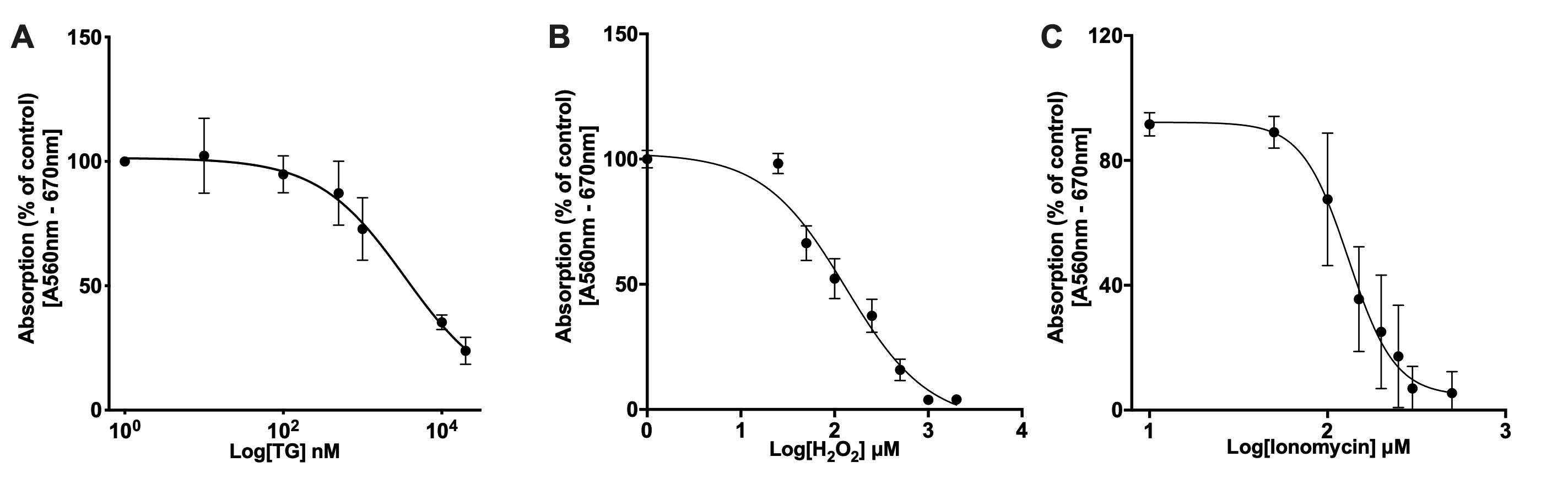

Supplement: Supplementary file 3 — Additional file 3: Figure S3. IC50 of Thapsigargin (TG), Hydrogen Peroxide (H2O2), and Ionomycin (Ion). HUVECs were subjected to different concentrations of TG, H2O2 and Ion for 4 h and MTT assay was performed. A HUVECs metabolic activity decreased with the increase of TG concentrations. Regression analysis was performed and IC50 of TG on HUVECs was found to be 3417 nM. B HUVECs metabolic activity also decreased with the increase of H2O2 concentrations. Regression analysis showed IC50 of H2O2 on HUVECs to be 131.3 μM. C HUVECs metabolic activity decreased with the increasing concentrations of Ion. Regression analysis yielded the IC50 of Ion on HUVECs as 117.4 μM. Data were represented as mean ± SD, n = 3, ns, not significant, *p < 0.05. [file 13287_2022_3228_MOESM3_ESM.tiff]

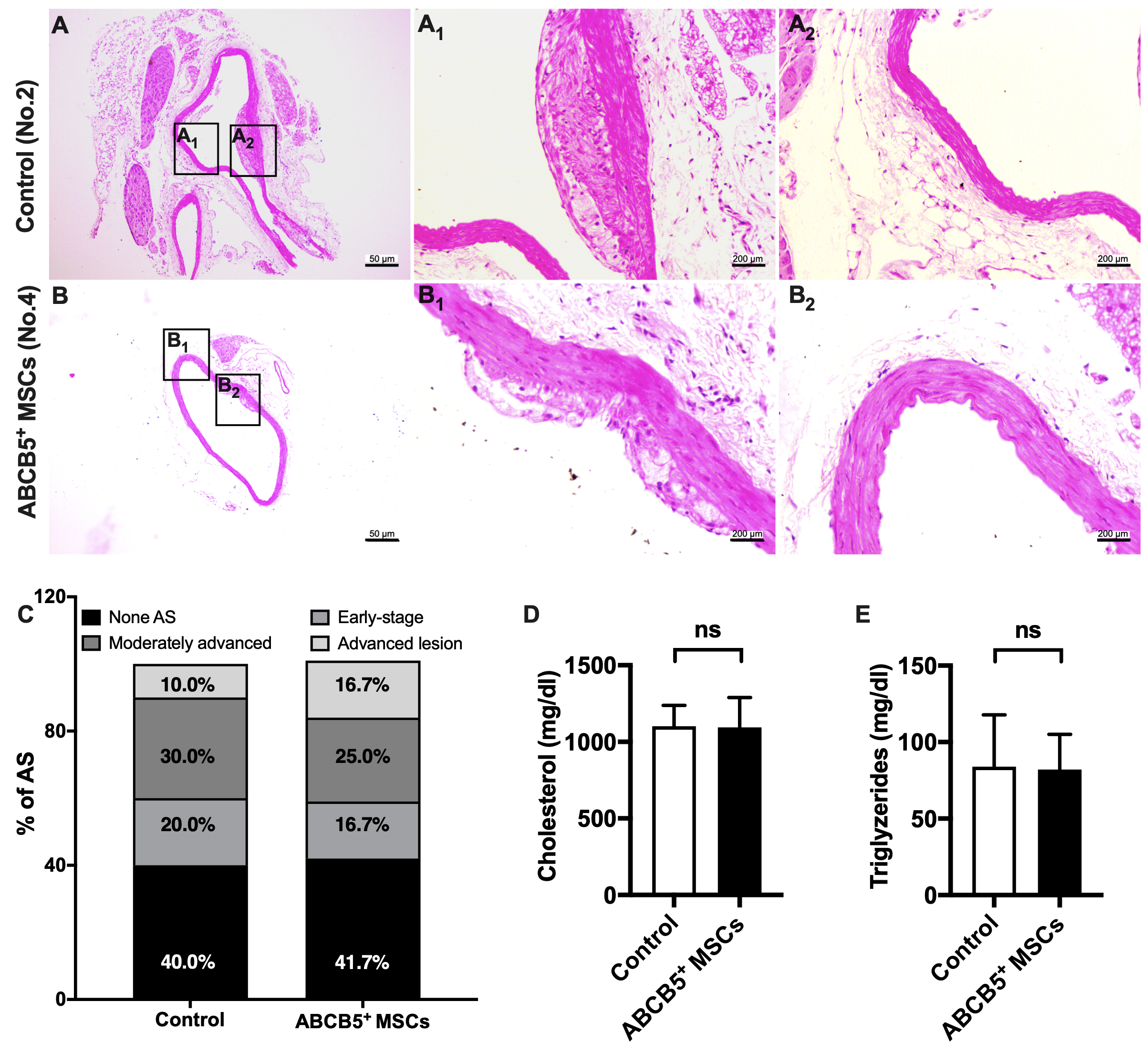

Supplement: Supplementary file 4 — Additional file 4: Figure S4. No significant difference was observed in AS lesions, cholesterol, triglycerides between ABCB5+ MSC treated and untreated mice. 7 days post DLFA, blood and aorta samples from mice were taken. A representative HE stained sections of the aorta from the A control group and B ABCB5+ MSC treated mice. A1 and B1 displays atherosclerotic vessel wall while A2 and B2 displays normal vessel wall (scale bar is 50 μm in Figure A and B; and 200 μM in Figure A1, A2, B1, B2). C Comparison of aortic AS lesion appearance between ABCB5+ MSC treated and control mice. D Triglyceride concentration and E Cholesterol concentration in plasma between two groups of mice. Data were represented as mean ± SD, n = 10 in ABCB5+ MSCs group, n = 12 in controls group, ns, not significant. [file 13287_2022_3228_MOESM4_ESM.tiff]

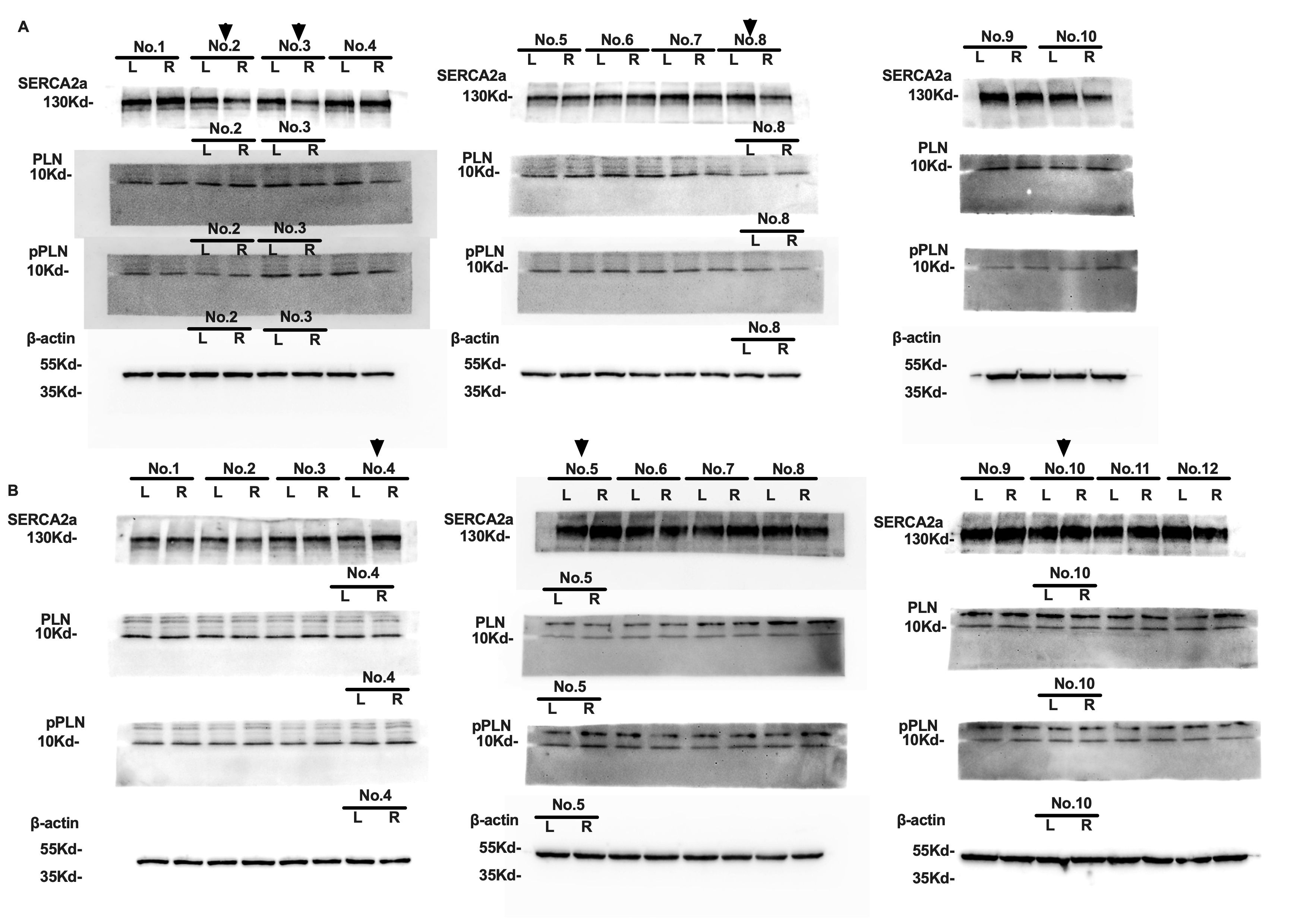

Supplement: Supplementary file 5 — Additional file 5: Figure S5. Full-length blots. A, B Full-length blots of SERCA2a, PLN, pPLN, and β-actin expression in left (L) and right (R) sides from control (A) and ABCB5+ MSC treated groups (B). [file 13287_2022_3228_MOESM5_ESM.tiff]
